# Interference with Hemozoin Formation Represents an Important Mechanism of Schistosomicidal Action of Antimalarial Quinoline Methanols

**DOI:** 10.1371/journal.pntd.0000477

**Published:** 2009-07-14

**Authors:** Juliana B. R. Corrêa Soares, Diego Menezes, Marcos A. Vannier-Santos, Antonio Ferreira-Pereira, Giulliana T. Almeida, Thiago M. Venancio, Sergio Verjovski-Almeida, Vincent K. Zishiri, David Kuter, Roger Hunter, Timothy J. Egan, Marcus F. Oliveira

**Affiliations:** 1 Laboratório de Bioquímica Redox, Programa de Biologia Molecular e Biotecnologia, Instituto de Bioquímica Médica, Universidade Federal do Rio de janeiro, Rio de Janeiro, Brazil; 2 Instituto Gonçalo Moniz, FIOCRUZ, Salvador, Brazil; 3 Departamento de Microbiologia Geral, Instituto de Microbiologia Prof. Paulo de Góes, Universidade Federal do Rio de Janeiro, Rio de Janeiro, Brazil; 4 Departamento de Bioquímica, Instituto de Química, Universidade de São Paulo, São Paulo, Brazil; 5 Department of Chemistry, University of Cape Town, Rondebosch, Cape Town, South Africa; Queensland Institute of Medical Research, Australia

## Abstract

**Background:**

The parasitic trematode *Schistosoma mansoni* is one of the major causative agents of human schistosomiasis, which afflicts 200 million people worldwide. Praziquantel remains the main drug used for schistosomiasis treatment, and reliance on the single therapy has been prompting the search for new therapeutic compounds against this disease. Our group has demonstrated that heme crystallization into hemozoin (Hz) within the *S. mansoni* gut is a major heme detoxification route with lipid droplets involved in this process and acting as a potential chemotherapeutical target. In the present work, we investigated the effects of three antimalarial compounds, quinine (QN), quinidine (QND) and quinacrine (QCR) in a murine schistosomiasis model by using a combination of biochemical, cell biology and molecular biology approaches.

**Methodology/Principal Findings:**

Treatment of *S. mansoni*-infected female Swiss mice with daily intraperitoneal injections of QN, and QND (75 mg/kg/day) from the 11^th^ to 17^th^ day after infection caused significant decreases in worm burden (39%–61%) and egg production (42%–98%). Hz formation was significantly inhibited (40%–65%) in female worms recovered from QN- and QND-treated mice and correlated with reduction in the female worm burden. We also observed that QN treatment promoted remarkable ultrastructural changes in male and female worms, particularly in the gut epithelium and reduced the granulomatous reaction to parasite eggs trapped in the liver. Microarray gene expression analysis indicated that QN treatment increased the expression of transcripts related to musculature, protein synthesis and repair mechanisms.

**Conclusions:**

The overall significant reduction in several disease burden parameters by the antimalarial quinoline methanols indicates that interference with Hz formation in *S. mansoni* represents an important mechanism of schistosomicidal action of these compounds and points out the heme crystallization process as a valid chemotherapeutic target to treat schistosomiasis.

## Author Summary

Heme is an essential molecule to most living organisms, but once in a free state it exerts toxic effects. Blood-feeding organisms evolved efficient ways to detoxify free heme derived from hemoglobin digestion. A key mechanism present in some hematophagous organisms consists of the crystallization of heme into a pigment named hemozoin. *Schistosoma mansoni* is one of the etiologic agents of human schistosomiasis, a parasitic disease that affects over 200 million people in tropical and subtropical areas. Hemozoin formation represents the main heme detoxification pathway in *S. mansoni*. Here, we report that the antimalarial quinoline methanols quinine and quinidine exert schistosomicidal effects notably due to their capacity to interfere with hemozoin formation. When quinine or quinidine were administered intraperitoneally during seven days to *S. mansoni*-infected mice (75 mg/kg/day), both worm and eggs burden were significantly reduced. Interestingly, hemozoin content in female worms was drastically affected after treatment with either compound. We also found that quinine caused important changes in the cellular organization of worm gastrodermis and increased expression of genes related to musculature, protein synthesis and repair mechanisms. Together, our results indicate that interference with hemozoin formation is a valid chemotherapeutic target for development of new schistosomicidal agents.

## Introduction

Schistosomiasis is a major parasitic disease that affects 170 million people in sub-Saharan Africa and close to 30 million people in north of Africa, Asia and South America [1]. Recent estimates indicate that 779 million people live in risk areas across 70 countries [2],[3],[4] raising the possibility that morbidity associated with the disease may be considerably under-estimated [5]. The disease pathology is typically a consequence of an inflammatory granulomatous reaction due to parasite egg deposition in the liver and other host tissues [6]. The major etiological agent of human schistosomiasis is the platyhelmith *Schistosoma mansoni* which is known to digest large amounts of blood in order to complete its development and sexual maturation [7]. During this process, host hemoglobin is degraded by several proteolytic enzymes [8],[9] forming peptides, amino acids and the prosthetic group heme [10].

Heme is an amphyphilic molecule of low molecular weight that plays essential biological roles, from cell respiration to drug detoxification [11]. A large body of evidence has demonstrated that once in a “free” state, heme is able to induce oxygen-derived free radicals formation [12],[13], lipid peroxidation [14],[15] and protein [16] and DNA [17] oxidation. Due to its amphyphilic nature, “free” heme also interferes with phospholipid membrane stability and solubility, in a mechanism independent of its pro-oxidant effects [18],[19], eventually resulting in cell lysis. As a consequence, it is apparent that blood-feeding organisms evolved efficient adaptations in order to circumvent the deleterious effects of “free” heme [20]. A particular mechanism present in some blood-feeders, such as shown in malaria parasites (*Plasmodium* sp.) [21], the kissing bug *Rhodnius prolixus*, a Chagas' disease vector [22] and the blood fluke *S. mansoni* consists of the crystallization of heme into a dark brown pigment known as hemozoin (Hz) [23]. Our group has shown that heme crystallization represents a major heme detoxification mechanism in both *R. prolixus* and *S. mansoni*, acting as a preventive antioxidant defense [24]. We have also shown that adult females of *S. mansoni* produce large amounts of Hz within the gut [23], involving extracellular lipid droplets present in the gut lumen in this process [25],[26]. Moreover, the hydrophilic-hydrophobic interface provided by the gut lipid droplets, seems to play a key catalytic role in heme crystallization, adding a strong biological support to the interface-mediated heme crystallization model recently proposed by Egan and co-workers [27].

Due to the essential nature of Hz formation in *Plasmodium* [27] the development of antimalarials throughout the years has been largely limited to the 4-aminoquinoline compounds, such as chloroquine (CLQ) and quinoline methanols such as quinine (QN) and quinidine (QND). These compounds exert potent action against the blood stages of *Plasmodium* in a mechanism that impairs Hz formation [28]. It was shown that 4-aminoquinolines interact with “free” heme, hindering its crystallization into Hz. The “free” heme interacts with membranes and exerts severe toxic effects, ultimately killing the parasite through oxidative stress [29]. An additional theory suggests that heme-quinoline complexes incorporate into a growing crystal face influencing its external appearance, and blocking its growth [30],[31]. Regardless of the mechanism by which 4-aminoquinolines act on Hz formation, our group has shown that CLQ inhibits heme crystallization *in vivo* in both *R. prolixus* [32], and also in *S. mansoni* [33],[26]. In this regard, we showed that *in vivo* treatment of *S. mansoni* -infected mice with CLQ decreased the overall severity of experimental murine schistosomiasis [33]. These results indicated for the first time that interfering with Hz formation in this parasite is a valuable approach for chemotherapeutic development. In addition, the Hz formation pathway is peculiar to blood-feeding parasites (including *S. mansoni*) and is absent in the host, which makes it an exceptionally attractive drug target. Very recent evidence indicates that the antimalarial mefloquine exerts potent antischistosomal effects, reducing worm burden [34], although its mechanism of action remains to be elucidated.

Currently, the main control strategy for schistosomiasis relies on chemotherapy [35], praziquantel (PZQ) being the main drug of choice for this purpose, as recommended by the World Health Organization. PZQ is safe, well tolerated and effective in a single oral dose treatment against the adult stages of all forms of schistosomiasis [36]–[38]. Despite the threat of resistance development of *S. mansoni* to PZQ [39]–[40], the establishment of true resistance so far is not conclusive [41]. Nevertheless, reliance on single PZQ therapy raises real concern and, as a result, this has prompted the search for new therapeutic targets and drugs against this disease. Other studies have suggested the antimalarial artemether as a new drug for schistosomiasis [42] due to its potent action against young schistosomula [43]–[45]. Alternative approaches, such as inhibition of the cysteine protease cathepsin B1 by K11777 early in the infection, have drastically decreased both worm and eggs burdens, delaying the egg-associated organ pathology [46]. The recent discoveries on the role of thioredoxin-glutathione reductase (TGR) activity for parasite redox balance and survival have prompted new chemotherapeutic development studies targeting this enzyme [47]–[49]. Inhibitors of *S. mansoni* TGR, such as oxadiazoles [49], and other drugs discovered in a massive screening [48], has provided new lead compounds that specifically inhibited parasite TGR with high potency, showing that an imbalance in the redox cascade is deleterious to *S. mansoni*, regardless of parasite stage [47]–[49].

Here we investigated the effects of the antimalarial quinolines QN, QND and QCR on *S. mansoni*-infected mice, by evaluating infection, biochemical, pathological, ultrastructural and molecular parameters.

## Methods

### Ethics statement

All animal care and experimental protocols were conducted following the guidelines of the institutional care and use committee (Comission for Evaluation of Animal Use for Research from the Federal University of Rio de Janeiro, CAUAP-UFRJ) and the NIH Guide for the Care and Use of Laboratory Animals (ISBN 0-309-05377-3). The protocols were approved by CAUAP-UFRJ under the registry #IBQM002. Technitians dedicated to the animal facility at the Institute of Medical Biochemistry (UFRJ) carried out all aspects related to the mice husbandry under strict guidelines to assure careful and consistent handling of the animals.

### Parasites and host animals

*S. mansoni* LE strain was maintained in the laboratory using *Biomphalaria glabrata* snails and Swiss mice as intermediate and definitive hosts, respectively. Cercariae released from snails were injected in mice cervices through a subcutaneous route. Mice were kept in a animal care facility at Institute of Medical Biochemistry (UFRJ). Forty-two days after infection, adult worms were obtained from the mice by mesenteric perfusion with saline as previously described [50].

### Regurgitant isolation

About 150 female adult worms were obtained by mesenteric perfusion of mice, placed in 1 mL of ultrapure water at room temperature for 80 minutes and gently shaken every 5 minutes. After that, the precipitated worms were discarded and supernatant, hereafter referred to as “regurgitant”, was colleted and kept at −70°C, as previously described by our group [26]. Protein content from regurgitant were measured relative to bovine serum albumin as a standard [51].

### Syntheses of new quinolines

The syntheses of compound 3 (C3), compound 6 (C6), compound 7 (C7), and compound 8 (C8) were accomplished by following a method previously described [52]. Synthesis of compound 9 (C9) was carried out using the protocol described in the literature [28]. For synthesis of compound 10 (C10), 4,7-dichloroquinoline (300 mg, 1.51 mmol) was added to a stirred solution of cyclopentylmethylamine (1.00 mL, 5.83 mmol) under N_2_. The mixture was heated under pressure in a cyclo-addition tube at 120°C overnight. After, the mixture was allowed to cool to room temperature, it was poured into saturated brine (20 mL) and extracted with ethyl acetate (3×50 mL). The organic layer was dried (Na_2_SO_4_) and concentrated *in vacuo* to afford a crude product, purified by flash column chromatography using a mixture of ethyl acetate: hexane (90∶10) as eluent to give C10 (295 mg, 87%) as a light-yellow solid, m.p. (EtOAc :Hex) 147–151°C; δ_H_ (CDCl_3_, 400 MHz) 8.51 (1H, d, *J* = 5.5 Hz), 7.94 (1H, d, *J* = 1.8 Hz), 7.63 (1H, d, *J* = 9.2 Hz), 7.32 (1H, dd, *J* = 1.8, 7.3 Hz), 6.43 (1H, d, *J* = 5.5 Hz), 5.05 (1H, br s), 4.01 (1H, m), 2.15 (2H, m), 1.80–1.61 (8H, m); δ_C_ (CDCl_3_, 100.5 MHz) 151.8, 149.3, 149.0, 134.8, 128.7, 125.2, 121.0, 117.2, 100.0, 54.3, 33.3.

### Heme crystallization inhibition assays

The assay utilized to evaluate inhibition of heme crystallization by quinoline antimalarials was based on a previous method described by Ncokazi *et al*, with minor modifications [53]. This method makes use of the known physico-chemical properties of heme that slowly and spontaneously crystallize into Hz particles *in vitro* in acidic conditions, in such a way that the reaction kinetics depends on the temperature, ionic strength, and time [54]. After reaction is complete, pyridine is added to the medium to promote the solubilization of the non-crystallized heme, since it is unable to solubilize heme within the Hz crystals. Then, aliquots of the supernatant were taken to determine the light absorption of non-crystallized heme at 405 nm. Samples corresponding to 15 µg of protein from whole regurgitant were incubated overnight at 37°C in 0.5 M sodium acetate buffer, pH 4.8, in the presence of 100 µM hemin (prepared in 0.1 N sodium hydroxide) in a 96-well plate with different concentrations of compounds (from 5 µM to 100 µM) in a total volume of 170 µL. Commercial quinolines and non-quinoline drugs were prepared in 100% ethanol whereas new quinolines were in 100% methanol. After that, 80 µL of 30.0% (v/v) pyridine solution in 20 mM Hepes sodium salt, pH 7.5 was added to each well, mixed and incubated for 15 minutes at room temperature, in order to allow sedimentation of Hz crystals. Then, 38 µL of supernatant were transferred to another 96-well plate, diluted to 250 µL in 30.0% (v/v) pyridine solution and the amount of free heme was determined using a microplate reader at 405 nm as previously described [53]. Hz content was determined by subtracting the amount of total heme added to the samples minus the values found in the experimental group. After that, data were plotted with GraphPad Prism 4.0 software and the IC_50_ was determined.

### Quinolines treatment *in vivo*

Female mice aged around 30 days were infected with *S. mansoni* by subcutaneous injection of 250 cercariae in the cervical region. Mice were treated in two different protocols, being the first one from day 11 to 17 after infection, with daily intraperitoneal injections of 75 mg/kg quinine hydrochloride dihydrate (QN), quinacrine (QCR) or quinidine (QND) prepared as 30.0% ethanol solutions. Control mice were treated with 30.0% ethanol alone (control). The volumes injected in each mouse was about 100 µL. Worms were collected by mesenteric perfusion 42 days after infection. In the second protocol, mice were treated from day 42 to 45 after infection with daily intraperitoneal injections of 100 mg/kg QND in 30.0% ethanol and the worms were collected by mesenteric perfusion 46 days after infection. The worms were separated by sex, counted and utilized for biochemical, ultrastructural and molecular assays. Mice small and large intestines, liver and plasma were collected for subsequent analyses. Non-infected mice were also treated with QN or 30.0% ethanol alone during the same period.

### Egg counting

Liver, small and large intestines of each mouse were collected after mesenteric perfusion, washed with phosphate buffered saline, weighed, sliced into small pieces, and subsequently digested overnight at 37°C in 10 mL of 4.0% potassium hydroxide (prepared in ultrapure water), as previously described [55]. Aliquots of digested tissues (10 µL) were placed onto a glass coverslip, and the number of eggs was determined by counting with a Zeiss stereomicroscope (Stemi SV11 MC80).

### Transmission electron microscopy (TEM)

Adult *S. mansoni* worms collected after mesenteric perfusion from mice were fixed in 1 mL of 2.5% glutaraldehylde and 4.0% formaldehyde in 0.1 M sodium cacodylate buffer, pH 7.4. The worms were fixed and post-fixed in 1.0% osmium tetroxide and 0.8% potassium ferricyanide in the same buffer, dehydrated in acetone and embedded in epoxy polybed resin. Thin cuts of the blocks were produced, contrasted in uranyl acetate and lead citrate and observed in a transmission electron microscopy Zeiss CEM 902, as described earlier by our group [25].

### Hz quantification

Hz in adult worms was extracted and quantified based on methods previously described by our group [23]. Worms were separated by sex, counted and homogenized in protease inhibitor cocktail (containing 0.1 mg/mL leupeptin trifluoroacetate salt, 2 mM benzamidine hydrochloride and 0.1 mg/mL soybean trypsin inhibitor in ultrapure water), hereafter referred as “homogenate”. For Hz extraction, 1 mL of female and male homogenates of *S. mansoni* were centrifuged at 15.000×*g* for 15 minutes at 25°C and then their supernatants were discarded. The pellets were washed three times in 1 mL of extraction buffer (contained a mixture of 0.1 M sodium carbonate and 2.5% sodium dodecyl sulphate), pH 9.1 and twice with 1 mL of ultrapure water. The final pellet was solubilized in 1 mL 0.1 N sodium hydroxide and shaken for 30 minutes. Hz content was determined spectrophotometrically at 400 nm in a GBC-UV/Vis-920 (Australia).

### Histopathology

Livers collected after mesenteric perfusion from control and QN-treated mice were washed with phosphate buffered saline, sliced into small pieces, fixed in 5 mL of 2.5% glutaraldehyde and 4.0% formaldehyde in 0.1 M sodium cacodylate buffer, pH 7.4 and kept at 4°C. Tissue slices were embedded in paraffin, stained with hematoxylin and eosin dyes and observed in an Olympus BX51 microscope. The mean granuloma area was analysed by using the ImageJ software available at the website (http://rsb.info.nih.gov/ij/).

### Microarray analysis

Freshly perfused adult female worms were incubated with cold RNA*later* solution (Ambion, USA) and kept at 4°C until RNA extraction. Total RNA was extracted using Trizol reagent (Invitrogen, USA) according to the manufacture's instructions. RNA was quantified using a Nanodrop ND-1000 UV/Vis spectrophotometer and its quality was assessed with an Agilent 2100 Bioanalyzer, a micro fluidics-based electrophoresis platform. 1 µg of total RNA from each sample was amplified using the T7-RNA polymerase based SuperScript Indirect RNA amplification system (Invitrogen, USA) and subsequently 3 µg of aminoallyl-modified amplified RNA was labeled using Cy3 or Cy5 reactive dye (GE Healthcare, USA). Amplification and labeling were done according to manufacturer specifications. Pools of RNA from worms treated with QN were hybridized against RNA from control worms. In order to ensure the uniformity of data, all experiments were performed using slides from the same printing batch and dyes from the same lot. Technical and biological replicates were performed. Dye swap was employed in order to account for dye biases. A total of eight different replicated data values were obtained for each condition and for each probe on the array. The Cy3- and Cy5-labeled samples were combined, dried and re-suspended in hybridization buffer (50% formamide, 25% RNase free water and 25% Microarray Hybridization buffer 4×); hybridization and washings were done according to the manufacturer GE Healthcare (USA). Samples were hybridized overnight to cDNA microarray slides containing 4,000 elements in duplicate (GEO accession: GPL3929) [56] using ASP hybridization chambers (GE Healthcare, USA) at 42°C. After washings, slides were air dried and scanned using a microarray dual channel laser scanner (GenePix 4000B, Molecular Devices, USA) at 5 µm resolution, 100% laser power and PMT levels were adjusted in order to obtain similar average intensities of red and green signal. Data were extracted using the program Array Vision 8.0. To correct for systematic biases on the data originated from small differences in the labeling and/or detection efficiencies between the fluorescent dyes, expression ratios were logged (base 2) and normalized using a locally weighted linear regression (LOWESS) algorithm [57]. Normalized log_2_ (ratios) were further analyzed with the Significance Analysis of Microarrays tool (SAM) [58] using a 0.1% false discovery rate (FDR) to find differentially expressed genes. Genes identified in the previous step were filtered using a 1.7 fold change cutoff in at least 4 out of the 8 data points. This step is critical in identifying biological relevant changes. The fold change filter was used after identification of significant differentially expressed genes, thus avoiding the bias caused by data trimming before significance testing [59]. Our microarray data was deposited in GEO with the accession number GSE14751.

### Aminotransferase assay

The activities of aspartate aminotransferase (AST) and alanine aminotransferase (ALT), indicators of hepatocellular damage, were measured in the plasma of control and QN-treated mice. Heparinized blood samples were obtained by cardiac puncture, centrifuged at 1.000×*g* for 10 minutes at room temperature and the plasma collected. AST and ALT activities were spectrophotometrically determined, as previously described [60].

### Determination of total thiol content

The assay utilized to quantify the total thiol content was based on a previous method described by Sedlack and Lindsay [61]. Aliquots of 50 µL of the total homogenates (corresponding to 150 µg of protein) were mixed with 150 µL of 200 mM TRIS buffer, pH 8.2, 10 µL of 10 mM DTNB (5, 5′-dithiobis-(2-nitrobenzoic acid) and 790 µL of 100% methanol. A reagent blank (without homogenate) and the sample blank (without DTNB) were prepared in a similar manner. The samples with or without DTNB were incubated at 25°C for 15 minutes. After that, samples were centrifuged at 3.000×*g* for 15 minutes at 25°C and the supernatants were used to estimate the thiols content by measuring light absorption at 412 nm in a GBC-UV/Vis-920. The total thiol content in the homogenate was determined by subtracting the values found in the experimental group minus the values found in the sample blank. The molar extinction coefficient of DTNB (13600 M^−1^ cm^−1^) was used to calculate the amount of DTNB reduced in each sample. The cellular content of thiol was plotted with GraphPad© Prism 4.0 software and expressed as nanomols of reduced DTNB/mg protein.

### Determination of lipid peroxidation by the thiobarbituric acid reactive substances assay (TBARS)

Samples corresponding to 120 µL of *S. mansoni* male or female homogenates were added to 180 µL sodium phosphate buffer, pH 7.4 plus 200 µL of 1.0% TBA in 50% acetic acid. The samples were incubated at 95°C for 15 minutes, cooled and added 500 µL *n*-butanol. The samples were centrifuged at 20.000×*g* for 10 minutes at room temperature and the absorbance of organic phase was measured spectrophotometrically at 532 nm in a GBC-UV/Vis-920. For quantification purposes, the millimolar extinction coefficient of malondialdehyde (MDA) of 156 mM/cm^−1^ was utilized.

### Assessment of intracellular reactive species

Female and male adult worms obtained by mesenteric perfusion of mice treated or not with QN were incubated with 1 µg/mL of 2′,7′-dichlorofluorescin diacetate (CMH_2_-DCFH-DA, Molecular Probes) in 200 µL of RPMI 1640 medium supplemented with 5% of fetal bovine serum, 2 mM of glutamine and 10 U.I/mL of penicillin and 10 mg/mL streptomycin for 30 minutes in the dark. After that, the worms were washed with saline solution and were observed in the fluorescence microscope (Axio Observer. Z1, Zeiss, Germany) using the excitation and emission wavelengths of 480 nm and 530 nm, respectively.

### *S. mansoni* culture

For cultivation of schistosomula, cercariae released from *Biomphalaria glabrata* snails were processed and converted to schistosomula by following a method previously described [62]. A water solution containing approximately 7200 cercariae was passed through a syringe needle (0.7 mm) four times and, after that, was left undisturbed for 90 minutes at room temperature to separate schistosomula from cercariae tails. In the laminar flow, the schistosomula pellet was washed five times in 5 mL of sterile saline solution containing 20 U.I/mL of penicillin and 20 mg/mL streptomycin, counted and transferred to 24 wells plates at a density of 150 schistosomula per well. Then, 2 mL of RPMI 1640 medium supplemented with 5% of fetal bovine serum, 2 mM of glutamine and 10 U.I/mL of penicillin and 10 mg/mL streptomycin was added in each well, followed by 2 µL of human red blood cells (hRBC). The culture was kept at 37°C and 4% CO_2_ and the medium supplemented with hRBC was changed everyday. Different concentrations of QN were added to the culture medium five days after starting the culture and was left for additional five days. In control group, ethanol was added to the culture medium to reach 0.1% of final concentration

Culture of adult worms was carried out by following the method described earlier with minor modifications [63]. Briefly, adult worms were obtained by mesenteric perfusion of mice with sterile saline, forty-two days after infection. In the laminar flow, the worms were washed three times with 5 mL of RPMI 1640 supplemented with 5% of fetal bovine serum, 2 mM of glutamine, 20 U.I/mL penicillin and 20 mg/mL streptomycin. A worm couple was plated per each well of a 24-wells plate in the presence of 2 mL of RPMI 1640 medium supplemented with 5% of fetal bovine serum, 2 mM of glutamine, 10 U.I/mL of penicillin and 10 mg/mL streptomycin. Human RBC (10 µL) was added to each well everyday and cultures were kept at 37°C and 4% CO_2_. The medium supplemented with hRBC was changed everyday. After two days of culture, QN was added to medium to reach 14.3 µM and 28.6 µM of final concentrations and kept for the following five days of culture. In control group, ethanol was added to the culture medium to reach 0.1% of final concentration. On the sixth day of culture the worms were collected and prepared for TEM as described above.

### Data analysis

The statistical analyses were done by non-paired *Student's* t test or one-way ANOVA analysis of variance and *a posteriori* Tukey's test for pair-wise comparisons. For all tests, a difference of *p*<0.05 was considered to be significant. *Student's t test*, ANOVA, Tukey's test and correlation analysis were performed by using GraphPad © Prism version 4.00 for Windows (GraphPad Software, USA).

## Results

### Quinoline compounds inhibit Hz formation induced by *S. mansoni* regurgitant *in vitro*

**Figure 1:**
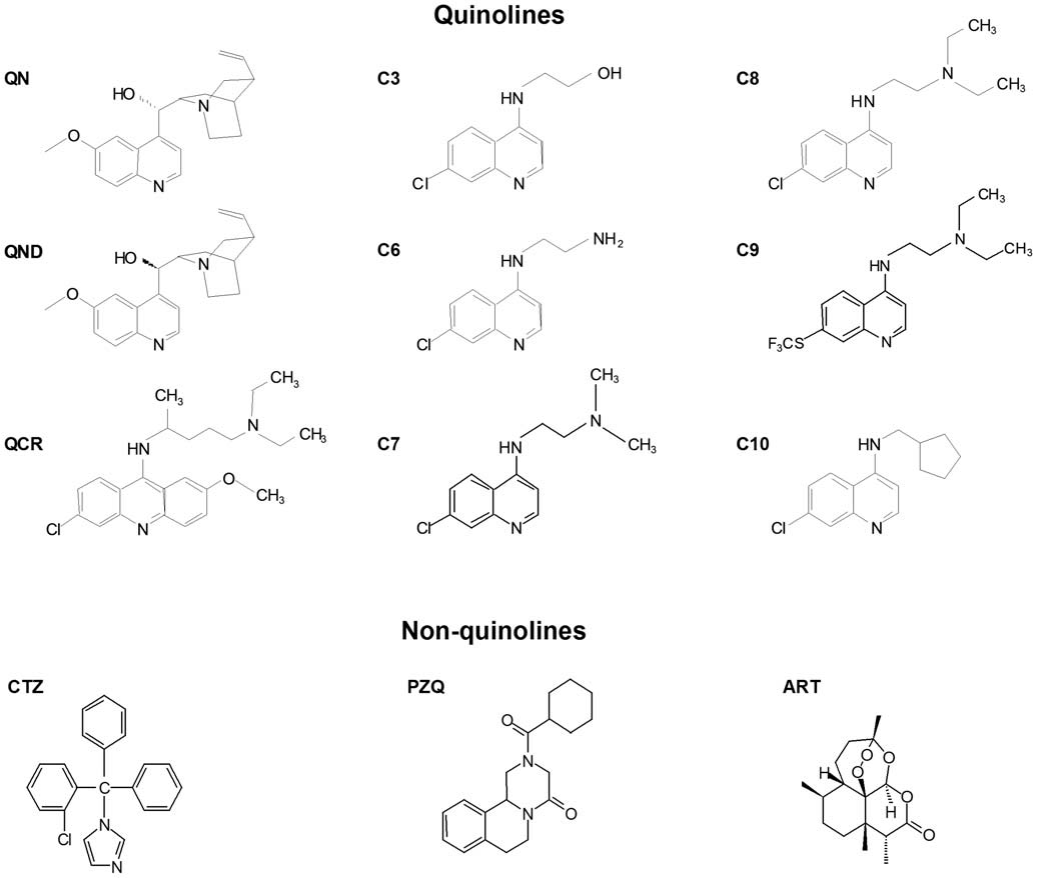
Chemical structures of quinoline and non-quinoline drugs. QN - quinine; QND - quinidine; QCR - quinacrine; C3 - compound 3; C6 - compound 6; C7 - compound 7; C8 - compound 8; C9 - compound 9; C10 - compound 10; CTZ - clotrimazole; PZQ - praziquantel; ART - artemisinin.

Although the mechanisms by which quinolines exert their antimalarial action are not completely understood, the capacity of these compounds to form complexes with heme *in vitro* and *in vivo* was already demonstrated [64],[65]. In fact, Kaschula and colleagues have shown that such antimalarial activity of quinolines normalized for pH trapping is directly correlated with their ability to inhibit Hz formation [28]. Previous evidence has indicated the schistosomicidal activity of some antimalarial compounds in murine models [33],[34],[66]. As demonstrated in table 1, a search over the literature revealed that from 11 antimalarial compounds tested so far in schistosomiasis models, 5 exhibited activity against *S. mansoni*. All these 5 compounds had their inhibitory action on heme crystallization reported elsewhere in chemically-driven heme crystallization (β-hematin formation) or in *Plasmodium* derived products-driven reactions. Therefore, our first attempt was to investigate the efficacy of quinoline and non-quinoline compounds to inhibit heme crystallization induced by *S. mansoni* regurgitant. Recent findings from our group demonstrate that, in *S. mansoni*, Hz is produced at the surface of extracellular lipid droplets (LD) found in the gut lumen and that enriched preparations of gut content, known as regurgitant, were able to produce Hz *in vitro* in reactions sensitive to quinolines [26]. We tested commercial quinolines such as QCR, QND and QN, new synthetic quinolines named C3, C6, C7, C9 and C10, and the non-quinolines artemisinin (ART), clotrimazole (CTZ) and praziquantel (PZQ) (Figure 1). Representative curves of inhibition of Hz formation by QN, QND and QCR are shown in Figure S1. We observed that all tested commercial quinolines efficiently inhibited heme crystallization, exhibiting the following IC_50_ values: QCR = 4.63 µM, QND = 2.41 µM and QN = 13.38 µM (Table 2). Also, synthetic C7 and C10 revealed to be potent inhibitors of this process, with IC_50_ values of 9.00 µM and 17.50 µM respectively (Table 2). Clotrimazole (CTZ) also showed great efficacy in inhibiting heme crystallization, with an IC_50_ value (10.22 µM) comparable with those of quinolines (Table 2). It was not possible to determine the IC_50_ values for ART, C3, C6 and C9 because no complete inhibition of heme crystallization was achieved up to the maximal concentration tested (100 µM). In addition, PZQ failed to interfere with heme crystallization, as previously demonstrated by our group [33], even in higher concentrations (1 mM). Thus, 9 out of 12 compounds tested, are known antimalarials and, from these, 8 inhibited reactions of β-hematin formation *in vitro*, as reported by the literature [28],[52]. Interestingly, all compounds tested in table 2 that inhibited in some extent Hz formation induced by *S. mansoni* regurgitant exhibit antimalarial effects and also interfere with β-hematin formation.

**Table 1:** Reported schistosomicidal activity and inhibitory effect on heme crystallization by antimalarial compounds.

Drugs	Schistosomicidal activity	Inhibitors of heme crystallization*
Amodiaquine	−a	+^b^
Chloroquine	+^a^	+^b^
Mefloquine	+^a^	+^b^
Quinine	+^a^	+^b^
Halofantrine	+^a^	+^b^
Pyronaridine	−a	+^b^
Lumefantrine	+^a^	+^c^
Sulfadoxine	−a	−
Sulfamethoxypyrazine	−a	−
Atovaquone	−a	−
Pyrimethamine	−a	−

a From Keiser *et al* [34].

b From Ncokazi and Egan [53].

c From Egan personal communication.

*: The reported inhibitory activity of antimalarial compounds on heme crystallization were obtained in synthetic reactions *in vitro*, and not in *Schistosoma*.

**Table 2:** Effect of different compounds on heme crystallization induced by *S. mansoni* regurgitant *in vitro*.

Drugs	IC_50_ (µM) Hz formation	Antimalarial activity	Inhibitor of βH formation
Quinacrine (QCR)	4.63±0.57	+	+
Quinidine (QND)	2.41±0.28	+	+
Quinine (QN)	13.38±0.82	+	+
Compound 3 (C3)	_	_	+^b^
Compound 6 (C6)	_	+	+^b^
Compound 7 (C7)	9.00±1.00	+	+^b^
Compound 8 (C8)	51.00±5.00	+	+^b^
Compound 9 (C9)	_	+	+^a^
Compound 10 (C10)	17.50±2.38	NT	+^c^
Artemisinin (ART)	_	+	_
Clotrimazole (CTZ)	10.01±2.22	+	+
Praziquantel (PZQ)	_	_	_

a From Kaschula *et al* [28].

b From Ncokazi and Egan [53].

c Egan personnal communication.

### Treatment of *S. mansoni*-infected mice with antimalarial quinoline methanols decreases worm burden and eggs production

**Figure 2:**
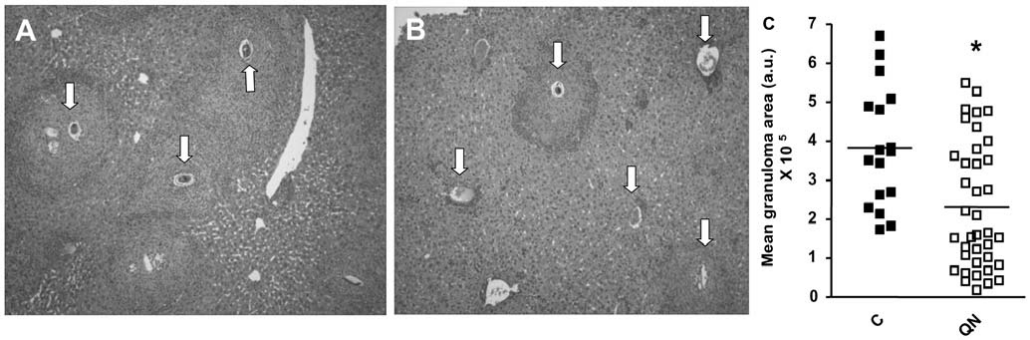
QN treatment decreases the granulomatous reaction to parasite eggs trapped in the liver parenchyma. Bright-field microscopy images of cross section of liver from control (A) and QN-treated (B) mice. Arrows indicate *S. mansoni* eggs deposited in mouse liver. Original magnification ×100. (C) Quantification of granuloma area in liver from control (closed squares, n = 17) and QN-treated (open squares, n = 38) mice (*p = 0.0021, Student's t test). Each symbol represents data from individual mice. The horizontal bars represent the median of granuloma area of each treatment.

Based on data shown in Table 2, we selected QN, QND and QCR for further tests of their potential schistosomicidal effects *in vivo* in a murine model. Due to the limited amounts of the synthetic compounds C7 and C10, *in vivo* assays with these compounds were not carried out. Then, in the first protocol tested, *S. mansoni*-infecetd mice received a daily intraperitoneal injection of QN, QND or QCR (75 mg/kg) over 7 days, beginning the treatment 11 days after infection. In this regime, QN administration was not toxic to mice (Figure S2A and S2B). During this period of drug administration the immature worm forms are usually located in the hepatic portal venous system, exhibiting developed and functional guts, and ingesting host blood to meet their nutritional demands. The worms were then recovered 42 days after infection by mesenteric perfusion of infected mice, a time point in which adult females are actively laying eggs that are frequently found in host liver, intestines and feces [67]. Table 3 shows that QN and QND treatment caused significant reductions not only in total worm (39% and 61%, *p*<0.001) but also in female worms burden (40% and 58%, *p*<0.001), respectively. QN treatment also caused a dose-dependent reduction in the viability of *in vitro*-transformed schistosomula kept in culture medium for five days (Figure S3). On the other hand, the acridine antimalarial QCR caused slight reductions in both total (24%) and female (18.5%) worms count (Table 3). QND was also tested by treating *S. mansoni*-infected mice with daily intraperitoneal injections of the drug (100 mg/kg) during 4 days, beginning the treatment 42 days after infection. Although in this regime QND was not toxic to mice (Figure S2C and S2D), there were no significant changes in the parasitemia (Figure S4A), viability (Figure S4B), and Hz content (Figure S4C). Table 4 shows that QN-treated mice had significant reductions in the eggs burden in the small intestine (65%, *p*<0.001), in the large intestine (89%, *p*<0.001), and also in the liver (42%, *p*<0.05). In QND-treated mice, significant reductions in egg burden were observed only in the small intestine (72%, *p*<0.05). Also, QCR treatment caused no significant inhibition of eggs deposition in all three tissues investigated (Table 4). Figure 2 shows that in QN-treated animals there were clear morphological changes in the granulomatous reaction surrounding the eggs deposited in the liver compared to control mice. In fact, QN treatment significantly decreased the size of granulomatous fibrotic foci which surround the eggs (39%, *p*<0.001) (Figure 2C).

**Table 3:** Effect of daily doses (75 mg/kg/day, intraperitoneal route from 11^th^ to 17^th^ day post infection) of two quinoline methanols and an acridine on worm burden to *S. mansoni*-infected mice, stratified by sex.

	No. mice	Mean number of worms per mouse (SEM)			Worm burden reduction (%)	
		Total	Male	Female	Total	Female
**Control**	51	101.7 (5.91)	60.68 (3.50)	43.38 (2.65)	-	-
**Quinine**	36	61.86 (4.79)^a^	41.77 (3.51)^a^	25.92 (2.58)^b^	39,17^a^	40,25^a^
**Quinidine**	8	39.50 (9.87)^a^	21.50 (5.43)^a^	18.00 (4.53)^c^	61,16^a^	58,50^b^
**Quinacrine**	6	76.83 (11.71)	41.50 (6.72)	35.33 (5.24)	24,45	18,55

Total number of *S. mansoni* worms was obtained after mesenteric perfusion of control, quinine, quinidine, and quinacrine-treated mice 42 days after infection. Statistical analyses between groups were performed by using ANOVA and *a posteriori* Tukey's test.

a p<0.001.

b p<0.01.

c p<0.05 compared to control.

SEM, standard error from mean.

**Table 4:** Effect of daily doses (75 mg/kg/day, intraperitoneal route from 11^th^ to 17^th^ day post infection) of two quinoline methanols and an acridine on eggs burden to *S. mansoni*-infected mice, stratified by tissue distribution.

	No. mice	Mean number of eggs deposited in mice tissues ×10^2^/g tissue (SEM)			Egg burden reduction (%)		
		Small intestine	Large intestine	Liver	Small intestine	Large intestine	Liver
**Control**	44	89.41 (11.95)	31.04 (7.35)	50.32 (5.70)	-	-	-
**Quinine**	40	30.53 (7.74)^a^	3.11 (1.41)^a^	29.17 (4.81)^c^	65,85^a^	89,98^a^	42,03^c^
**Quinidine**	8	24.84 (10.90)^c^	0.62 (0.35)	23.09 (7.64)	72,22^c^	98,00	54.11
**Quinacrine**	6	48.09 (10.47)	4.93 (1.77)	33.39 (12.01)	46,21	84,11	33,64

Total number of *S. mansoni* eggs was obtained after mesenteric perfusion of control, quinine, quinidine, and quinacrine-treated mice 42 days after infection. Statistical analyses between groups were performed by using ANOVA and *a posteriori* Tukey's test.

a p<0.001.

c p<0.05 compared to control.

SEM, standard error from mean.

### Antimalarial quinoline methanols affect *in vivo* heme crystallization in *S. mansoni*

**Figure 3:**
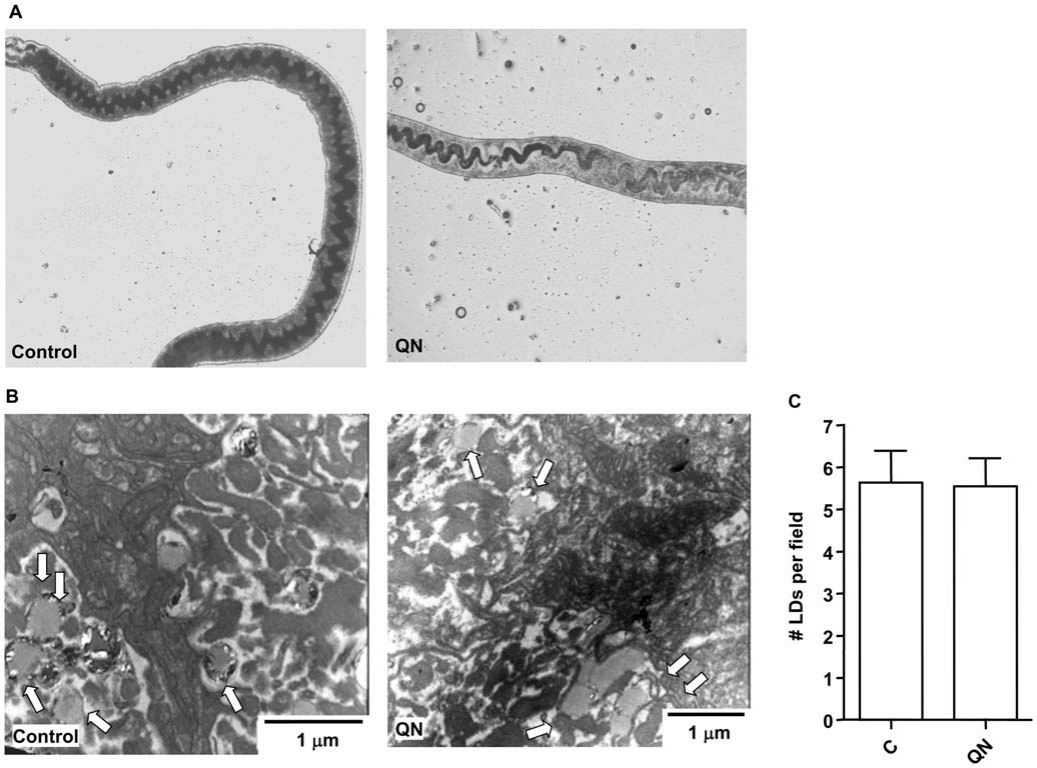
QN treatment decreases Hz content on *S. mansoni* females *in vivo*. (A) Bright field microscopy images of control and QN-treated (QN) females of *S. mansoni*, showing clearly a reduction in the pigment content of the gut. Original magnification ×25. Control: *S. mansoni*-infected mice. QN: *S. mansoni*- infected mice treated with 75 mg/kg/day QN. (B) TEM of cross sections of gastrodermis from control and QN-treated (QN) *S. mansoni* females. The arrows indicate the lipid droplets found in the gut lumen. (C) Quantification of LDs in the gut of control (C, n = 12) and QN-treated (QN, n = 28) female worms obtained from TEM images at 12.000×magnification. Results are means±SEM. Bars denote the scale in micrometers.

**Figure 4:**
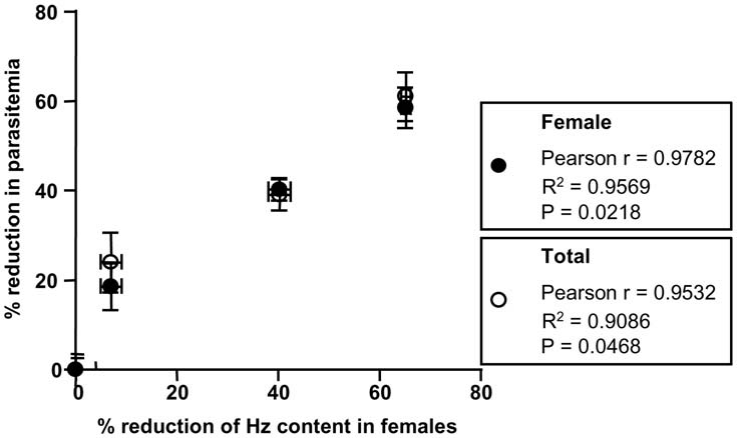
Relative reduction in female parasitemia correlates with reduction of their Hz content. Circles represent values of both percentage of reduction in total parasitemia as well as the percentage of reduction in Hz content in females after treatment with 75 mg/kg/day QN, QND or QCR from day 11 to 17 after infection. Black circles represent the data from female worms whereas white circles were from total worms. Values of correlation analyses are expressed in the graph.

Aiming to investigate whether the effects of QN, QND and QCR treatment on *S. mansoni* were related to the impairment of heme crystallization process, Hz content was determined in both male and female worms using previously established methods [23]. The reduction of Hz content in the gut of QN-treated females was clearly visible when observed by bright field microscopy (Figure 3A). QN treatment also caused a clear reduction of Hz crystals present on the surface of LDs in the gut lumen of *S. mansoni* females (Figure 3B, *arrows*). Since Hz formation in *S. mansoni* occurs at the surface of LDs [26], one could argue that reductions in the amount of crystals found in QN-treated worms could be a consequence of less LDs present in the gut lumen. This seems not the case since an inspection of TEM images showed that there were no changes in the number of LDs observed in female's gut caused by QN treatment (Figure 3C). In fact, both QN and QND significantly decreased Hz content in female worms by 40% and 65%, respectively (Table 5). Curiously, reductions in the Hz content were less pronounced in male worms treated with both drugs (17% to 21%) compared to females. Also, QCR treatment caused slight reductions in Hz content in both male and female worms (7% to 25%). Interesting to note is that relative reductions in both total and female parasitemia strongly correlated (R^2^ = 0.90 to 0.95, *p*<0.05) with the relative reduction in the Hz content found in female worms (Figure 4). In addition, QN, QND and QCR treatment did not cause any changes in the total protein content of both male and female worms (Figure S5). Curiously, inhibition of heme crystallization did not result in redox imbalance in adult worms since the content of total reduced thiols, as well as the levels of lipid peroxidation products remained unchanged in QN-treated *S. mansoni* females (Figure S6). Finally, fluorescence microscopy analyses revealed that in QN-treated worms the levels of reactive species is undistinguishable from control ones assessed by the fluorescent reactive probe CMH_2_-DCFDA (Figure S7).

**Table 5:** Effect of daily doses (75 mg/kg/day, intraperitoneal route from 11^th^ to 17^th^ day post infection) of two quinoline methanols and an acridine on hemozoin content in *S. mansoni*, stratified by sex.

	No. mice	Mean hemozoin content in worms as nmols heme/mg protein (SEM)		Reduction in Hz content (%)	
		Male	Female	Male	Female
**Control**	27	2.48 (0.35)	36.67 (3.97)	-	-
**Quinine**	27	1.95 (0.20)	21.90 (2.19)^a^	21.37	40.28^c^
**Control**	8	1.12 (0.19)	19.86 (1.52)	-	-
**Quinidine**	6	0.93 (0.30)	6.92 (0.91)^a^	17.31	65.16^a^
**Quinacrine**	6	0.84 (0.19)	18.46 (2.07)	25.54	7.05

Hemozoin content was obtained from *S. mansoni* worms recovered after mesenteric perfusion of control, quinine, quinidine, and quinacrine-treated mice 42 days after infection. Statistical analyses between groups were performed by using ANOVA and *a posteriori* Tukey's test.

a p<0.001.

c p<0.05 compared to control.

SEM, standard error from mean.

### Quinine treatment causes remarkable ultrastructural changes in the gastrodermis of adult worms

**Figure 5:**
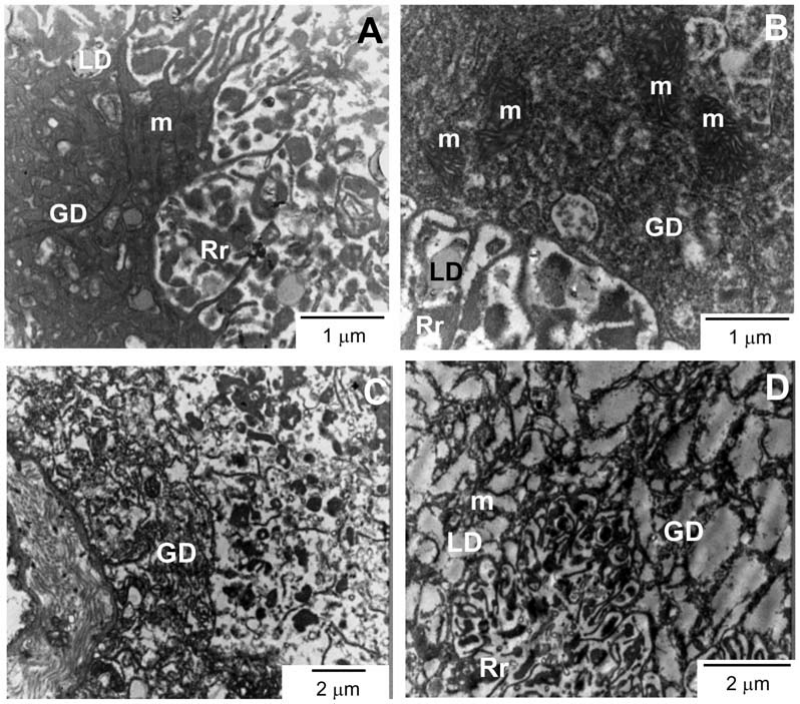
QN treatment *in vivo* causes remarkable ultrastructural changes in the gastrodermis of adult worms. TEM images of cross sections from adult females (A, B) and males (C, D) gastrodermis. Panels A and C were from control mice, while panels B and D were from QN-treated mice. GD-gastrodermis; LD- Lipid droplets; Rr- Red blood cells remnants; m- mitochondria. Bars denote the scale in micrometers.

**Figure 6:**
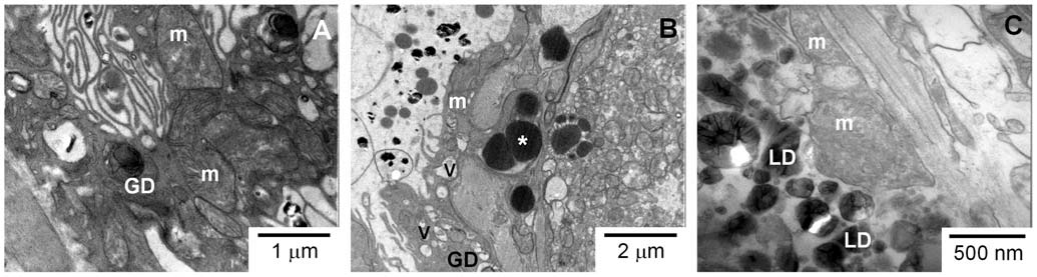
QN treatment affected the gastrodermis of adult worms cultured *in vitro*. TEM images of gastrodermis cross sections from adult females of *S. mansoni* cultured *in vitro* for six days. Panel A represents female worms treated with 0.1% ethanol for 48 h. Panel B represent female worms treated with 14.3 µM QN for 48 h. Panel C represents female worms treated with 14.3 µM QN for 72 h. GD-gastrodermis; LD- Lipid droplets; m- mitochondria; V-vacuoles. Asterisks indicate electron-dense particles within gastrodermis cells. Bars denote the scale in micrometers or nanometers.

In an attempt to investigate whether inhibition of heme crystallization promoted by QN treatment affected parasite ultrastructure, we observed control and QN-treated adult worms by transmission electron microscopy (TEM) as shown in figure 4. An initial inspection of different parasite regions indicated that QN indeed caused ultrastructural modifications in *S. mansoni*. Curiously, we did not observe substantial alterations in the tegument and musculature of both female (Figure S8A and S8B) and male (Figure S8C and S8D) worms recovered from QN-treated mice. More importantly, detailed analyses indicated that QN treatment caused remarkable ultrastructural changes in the gastrodermis of both female (Figure 5A and 5B) and male worms (Figure 5C and 5D). Noteworthy is the extensive loss of electron density and clear indications of both cytoskeleton disorganization and mitochondrial swelling observed in female gastrodermis (Figure 5B). Regarding the male worms, the most striking change observed in QN-treated worms was the washed-out aspect of the cytosol of gastrodermis cells (Figure 5D). In addition, mitochondria were found inside autophagic vacuoles in the gastrodermis (Figure S9A) as well as with swollen appearance, washed-out matrix and with remnants of inner membranes in the sub-tegumentar region of QN-treated female worms (Figure S9C). Finally, adult females cultured *in vitro* in the presence of QN (14.3 µM) for 48 h promoted gastrodermis vacuolization and the appearance of huge electrondense particles within the tissue (Figure 6B). Interestingly, QN caused a complete destruction of females gastrodermis after 72 h of treatment as shown in figure 6C where LD usually found in the gut lumen were found in close association in mitochondria, possibly derived from the gastrodermis.

### Quinine treatment increased expression of transcripts related to sexual differentiation, musculature, cytoskeleton and repair mechanisms in *S. mansoni* females

In order to characterize the effect of QN treatment at the molecular level, we performed large-scale gene expression analyses using cDNA microarrays with approximately 4,000 different *S. mansoni* gene fragments (GEO accession number: GPL3929) [58]. Gene expression levels were compared between female adult worms recovered from infected mice that were treated with QN and female worms recovered from control (non-treated) infected mice. We have found 25 transcripts significantly up-regulated in QN treated parasites (Figure S10). These transcripts were mapped to the draft genome sequence and gene predictions generated by the *S. mansoni* Genome Project [68] (Table 6). Additional annotations were obtained by similarity searches against the NCBI nr database (Table 6). A considerably high proportion (6/25 = 24%) of the up-regulated genes is composed of muscle-related genes, including myosin heavy and regulatory chains (C604749.1, C607128.1 and C600026.1); tropomyosin (C608351.1); troponin (C605852.1) and titin (C609457.1). Moreover, the expression of two different kinases was induced after QN treatment: a src tyrosine kinase (TK3) (C600304.1) and a SNF-1 related kinase (SNRK) (C603981.1). Over-expression of a GPI-anchored surface glycoprotein (C601690.1), described to be critical for PZQ effectiveness [69], was detected in QN-treated parasites. Increased expression of GPI-anchored surface glycoprotein was confirmed by real-time PCR experiments (data not shown). Based on the data shown here, the most significant changes observed in parasite gene expression promoted by QN treatment were the increased expression of genes related to repair mechanisms: aldehyde dehydrogenase (ADH) (C609066.1) and rad25/xp-B DNA repair helicase (C704554.1). We have also detected an increased expression of aminophospholipid flippase (or translocase) (C605890.1), which is involved in prevention of apoptosis by avoiding phosphatidylserine externalization on cell surface [70],[71]. Activation of these genes may represent a key strategy to avoid recognition of damaged parasite cells by the host immune system.

**Table 6:** Genes most differentially expressed in *S. mansoni* adult female promoted by QN treatment.

Read	Contig	Predicted gene Genome project	Gene annotation Genome project	GI	ACC	BLAST hit annotation	Mean log2Ratio	Mean Fold change
MG1-0081G-D263-C03-U.B	C603095.1	none	none	none	none	No hits	0,87	1,83
MS1-0091T-D221-E01-U.G	C607687.1	Smp_142100	Transcriptional cofactor CA150, isoform 4	114431215	CAK32514.1	transcriptional cofactor CA150 [*S. mansoni*]	0.74	1.68
MM1-0020T-R029-C08-U.G	C603981.1	Smp_068990	SNF-1 related kinase (snrk)	108880563	EAT44788	serine/threonine protein kinase [*Aedes aegypti*]	0.66	1.58
MS1-0010T-D100-C07-U.G	C605852.1	Smp_179810	Troponin t	none	none	No hits	0.79	1.73
MS1-0060P-V267-F08-U.B	C609534.1	none	none	none	none	No hits	0.74	1.67
MG1-0012T-L304-G02-U.B	C704654.1	Smp_165580	rad25/xp-B DNA repair helicase	115927405	XP_79417.2	similar to DNA repair gene [*Strongylocentrotus purpuratus*]	0.61	1.53
ML1-0094T-D143-A12-U.G	C603809.1	Smp_143140/Smp_143150	Eukaryotic translation elongation factor	147903679	NP_001086877.1	translation elongation factor 2 [*Xenopus laevis*]	1.09	2.13
MG1-0024U-A221-C05-U.B	C600026.1	Smp_085540	Myosin heavy chain	none	none	No hits	1.09	2.13
MA3-0001U-M322-C04-U.G	C609457.1	Smp_105020	Titin (contain immunoglobulin domain)	none	none	No hits	0.63	1.55
ML1-0070T-M249-C11-U.G	C602410.1	Smp_144800	ormdl protein	57529367	NP_001006288.1	Ribophorin II [*Gallus gallus*]	0.58	1.50
MS1-0051T-L291-B10-U.B	C603837.1	Smp_173670	Expressed protein	none	none	No hits	0.80	1.74
MA1-0040P-L070-B02-U.B	C610344.1	Smp_161910	hypothetical protein	56758142	AAW27211.1	SJCHGC09295 protein [*Schistosoma japonicum*]	0.92	1.89
ME1-0006T-L093-F08-U.B	C612031.1	Smp_180010	hypothetical protein	91086677	XP_968541.1	Similar to Vac14 homolog [*Tribolium castaneum*]	0.63	1.55
MS1-0053T-L279-C06-U.B	C601690.1	Smp_017730/Smp_131910	GPI-anchored surface glycoprotein	none	none	No hits	0.51	1.43
ML1-0055P-A137-B03-U.B	C600904.1	Smp_099870/Smp_189530/Smp_020460/Smp_135320	Elongation factor 1-alpha	1619614	CAA69721.1	elongation factor 1-alpha [*Schistosoma mansoni*]	0.63	1.55
MG1-0104U-A348-H01-U.G	C604892.1	Smp_027850	Expressed protein	60698352	AAX30948.1	SJCHGC09258 protein [*Schistosoma japonicum*]	0.65	1.57
MA3-0001U-M318-A10-U.B	C607128.1	Smp_038440	heart-specific myosin light chainphosphatase small subunit	110778182	XP_624583.2	Myosin binding subunit [*Apis mellifera*]	0.60	1.52
MA3-9999U-L241-F07-U.G	C605890.1	Smp_104500	phospholipid-transporting atpase-related (aminophospholipid flippase)	149703022	XP_001494366.1	similar to ATPase II [*Equus caballus*]	0.53	1.44
MA1-0053U-V063-C06-U.B	C600304.1	Smp_151300	proto-oncogene tyrosine-protein kinase src	37776869	CAE51198.1	src tyrosine kinase [*Schistosoma mansoni*]	0.54	1.45
MG1-0068P-V304-A05-U.B	C602627.1	Smp_141610	cathepsin B endopeptidase	18181863	CAC85211.2	cathepsin B endopeptidase [*Schistosoma mansoni*]	0.54	1.46
MG1-0068G-V328-C09-U.B	C601156.1	Smp_034940	Protein C10orf118 (CTCL tumor antigen HD-CL- 01/L14-2)	2623840	AAB86568.1	unknown [*Schistosoma mansoni*]	0.53	1.44
MG1-0070T-D265-C04-U.B	C608351.1	Smp_044010	Tropomyosin	1174754	P42637	Tropomyosin I [*S. mansoni*]	0.55	1.46
MA3-0001U-M327-C07-U.G	C605169.1	Smp_030300	Thrombospondin	none	none	No hits	0.68	1.61
MG1-0046U-A225-E02-U.B	C604749.1	Smp_132670/Smp_132680	Myosin regulatory light chain	none	none	No hits	0.66	1.58
MA3-0001U-M340-C04-U.G	C609066.1	Smp_133510	Aldehyde dehydrogenase	76155963	AAX27215.2	SJCHGC03451 protein [S. japonicum]	0.64	1.56

## Discussion

We reported here that interference with heme crystallization in *S. mansoni* represent an important mechanism by which antimalarial quinoline methanols exert their schistosomicidal action. These compounds caused significant reductions in both worm and egg burden, whereas remarkable changes in the ultrastructural organization of parasite gastrodermis, as well as induced expression of genes related to musculature, protein synthesis and repair mechanisms were seen in worms treated with QN. From all the three compounds tested, QN and QND caused the most striking reductions in the Hz content on female worms, which are more susceptible to interference in this process [33]. Interestingly, reduction in total and female parasitemia, obtained after treatment with QN, QND and QCR, was positively correlated with the percentage of Hz content reduction in female worms (Figure 3), strenghtening the concept that interference with heme crystallization plays an important role on the mechanism of schistosomicidal action of antimalarial quinoline methanols. The present work, together with previous evidence describing schistosomicidal activity of antimalarial quinolines [33],[34],[66], strongly indicates that heme crystallization in *Schistosoma* is a promising target for the development of new schistosomicidal compounds.

Hz formation represents the main heme detoxification mechanism in *S. mansoni*, accounting for more than 50% of total heme content in female worms [23]. Our group firstly proposed that interference in Hz formation could be a potential target for new therapies against schistosomiasis [33] by showing that treatment of *S. mansoni*-infected mice with multiple intraperitoneal injections of CLQ caused an important decrease in the overall severity of disease. In this regard, it is important to notice some aspects related to the results of inhibition of heme crystallization by antimalarial quinoline methanols obtained in the present work, as following: *i)* Hz content observed in female worms after QN and QND treatment were normalized by the worm's protein content (Table 5), a parameter that was not affected after quinoline methanols treatment (Figure S5). Thus, reductions in the Hz content observed (Table 5) were not related to reduced worm counts (Table 3) or to increased protein levels found upon treatment; *ii)* since the LDs found in the gut lumen are responsible for Hz production in *S. mansoni* [26] reduced Hz formation (Table 5) is not a consequence of less LDs found in the gut lumen, since treatment with antimalarial quinoline methanols did not affected LD counts (Figure 3C); *iii)* the relative reduction in parasitemia was positively correlated with the relative reduction of Hz content in females (Figure 4), which, once again strongly denotes the role of heme crystallization to parasite survival; *iv)* previous evidence from the literature, demonstrating schistosomicidal activity of antimalarial drugs, show a high agreement with those compounds that also inhibit synthetic β-hematin formation (Table 1). The only exception is amodiaquine, since the drug interferes with heme crystallization *in vitro*, although did not cause any change in parasitological parameters described by Keiser and collagues [34] (Table 1). Conceivably, the metabolic processing of amodiaquine, and its short plasmatic half-life (5.2 minutes), may affect not only the drug accucmulation inside *Schistosoma* gut, but also the final concentrations necessary to inhibit Hz formation, ultimately failing to cause parasite death [34]; *v)* in table 2, from 12 compounds tested, 9 are known antimalarials and, from these, 8 are known inhibitors of β-hematin formation *in vitro*, as reported by the literature [28],[52]. Interestingly, 6 out of these same 8 compounds inhibited Hz formation induced by *S. mansoni* regurgitant with different intensities. Therefore, although alternative explanations can be raised, interference with Hz formation seems to be the simplest explanation for the findings presented here.

Several reports proposed the relevance of a chlorine atom at 7-position of the quinoline ring as an important structural determinant for the inhibitory effects on heme crystallization *in vitro* [28],[53]. Despite its obvious relevance, this feature alone is not sufficient to determine their antimalarial effect because another important aspect must be considered. Drugs accumulation within the *Plasmodium* food vacuole play also a key role since this allow quinoline compounds reach intracellular concontrations necessary to inhibit with Hz formation. In fact, Kaschula and colleagues obtained an excellent correlation between antiplasmodial activity, normalized for pH trapping, and beta-hematin inhibitory activity [28]. In this regard, despite the lower IC_50_ values obtained *in vitro*, neither Hz formation *in vivo* nor parasite burden were affected by QND when it was administered later on infection (Figure S4). However, reduction in both Hz content in female worms (Table 5), as well as parasite and eggs burden were achieved when QND was administered early on the infection (Tables 3 and 4), indicating that interference with heme crystallization is an important aspect to determine its schistosomicidal effects. Also, QCR treatment caused no changes in worm burden and Hz formation as well (Tables 3 and 5). Thus, based on the data presented here, we cannot exclude the possibility that drug accumulation, through pH trapping, could be also determining some of the schistosomicidal effects of antimalarial quinoline methanols. Since these compounds are weak bases, it is possible that their accumulation may affect the parasite gut pH and then interfering with parasite proteolytic enzymes. In fact, Bogitsch and Davenport demonstrated that lysosomotropic agents, such as chloroquine, accumulates within *S. mansoni* schistosomula cultured *in vitro* [72]. Further research on these aspects will provide a better understanding on the contribution of these factors in determining the schistosomicidal action of quinolines.

Praziquantel (PZQ) administration represents the main strategy for schistosomiasis control worldwide, but the threat of resistance development to PZQ has prompted the search for new schistosomicidal compounds [39]. In this sense, quinoline compounds have been used as the main therapeutic option for malaria treatment for more than 300 years [73]. Their wide use in the treatment of malaria is in part due to their high safety and efficacy, in addition to their well known pharmacokinetics. Consensus over the mechanism of antimalarial action of CLQ suggests that it relies on its interaction with heme [64] by forming a complex that accumulates within the acidic food vacuole [74] which impairs heme crystallization therein [75]. The complex formed between heme and quinolines increased heme affinity for biological membranes, enhancing its toxic effects [29],[74],[75] and also inhibiting heme crystallization in *Plasmodium* [76],[77],[78]. Possibly, heme molecules derived from hemoglobin digestion would be diverted from their natural fate (Hz) by forming a stable complex with quinoline methanols, which, in turn, would increase their association with hydrophilic-hydrophobic interfaces present not only in LD but also in other biological membranes, such as those of the gastrodermis cells (Figures 3B, 5B, 5D and 6C). Moreover, increased association of heme-QN complex with the plasma membrane of gastrodermis cells would explain the remarkable cytoskeleton disorganization observed in QN-treated worms (Figures 3B, 5B, 5D and 6C), caused by heme-induced changes in membrane stability and oxidative modifications. An interesting observation in table 2 is that PZQ caused no effect on heme crystallization *in vitro*, reinforcing the potential of therapeutic regimens combining PZQ with antimalarial quinoline methanols, as these compounds affect distinct metabolic pathways. Further research is necessary to determine whether this combined treatment turns out to be a valuable clinical strategy for schistosomiasis chemotherapy.

Quinoline methanols administration did not result in complete elimination of parasites from *S. mansoni*-infected mice, and a possible explanation could be the induction of genes that enable parasite survival upon impaired heme crystallization. Changes observed in parasite gene expression would be a consequence of increased levels of non-crystallized heme complexed to quinoline methanols. Noteworthy, QN-exposed parasites showed up-regulation of 25 genes (Table 6), which can be grouped into 8 categories: sexual differentiation, musculature, cytoskeleton, signal transduction, transcription/translation, protein digestion, recognition, and repair mechanisms. Curiously, among the induced genes was TK3 (probe C600304.1), a gene encoding a src tyrosine kinase predominantly expressed in the reproductive organs, as well as in female's vitellarium [79], being involved in signal transduction pathways related to the cytoskeleton in Schistosoma gonads [79]. TK3 is a herbimycin probable target, having key functions in regulating gonad development and egg production [80]. Over-expression of a kinase with putative cell-architecture regulatory function may indicate that a cytoskeleton reorganization process takes place as part of an effective survival response to QN administration.

In QN-treated worms we have detected over-expression of a gene encoding a GPI-anchored surface glycoprotein (probe C601690.1), described to be critical for PZQ effectiveness [69]. It has been postulated that antibodies that develop against some unique glycoproteins exposed at the parasites' tegument surface are important for an effective action of PZQ [42], as PZQ effectiveness is impaired in immune-deficient animals [42]. In fact, monoclonal antibodies that recognize the GPI-anchored surface glycoprotein are able to restore PZQ efficacy in B-cell depleted mice [69]. It is tempting to hypothesize that the observed QN-induced over-expression of the tegument glycoprotein gene could favor an augmented action of PZQ, thus favoring the idea of a QN-PZQ combined treatment; since the threat of PZQ resistance development represents a real concern, QN would be a good PZQ partner for schistosomiasis treatment.

Despite the schistosomicidal effects of quinoline methanols, direct evidence linking this property to increased oxidative stress conditions, due to impaired heme crystallization in parasite gut (Figure 3B, and Table 5) remains inconclusive. Measurements of total reduced thiol content (Figure S6A) as well as lipid peroxidation products (Figure S6B) and the assessment of reactive species in QN-treated worms by using the fluorescent probe CMH_2_-DCFA (Figure S7), failed to demonstrate changes in these parameters. We speculate that the absence of oxidative stress markers in QN-treated parasites could be explained by an increased expression of genes related to repair mechanisms. In fact, we observed that 4 out of 25 genes over-expressed in QN-treated worms were related to mechanisms involved in cellular repair and maintenance (Table 6). Noteworthy is the increased expression of aldehyde dehydrogenase (ADH) gene, which encodes an enzyme involved in detoxification of lipid peroxidation products such as reactive malondialdehyde and hydroxynonenal [81], and of rad25/xp-B DNA repair helicase gene. Increased expression of both ADH and rad25/xp-B DNA repair helicase genes would explain the preserved ultrastructure in surviving parasites recovered at the end of the treatment period. Further, ultrastructural evidence (Figure S9A) indicates that mitochondrial autophagy in the gastrodermis of QN-treated female worms would play a cellular protective role by eliminating dysfunctional organelles and preventing mitochondrial-induced apoptosis.

In conclusion, the results presented here show that interference with heme crystallization in *S. mansoni* by quinoline methanols exerts promising schistosomicidal effects, causing significant reductions in several pathologic parameters of disease. Since the endemic areas of malaria and schistosomiasis overlap in many regions of the globe and, considering that Hz formation occurs both in *Plasmodium* and *Schistosoma* parasites, retrospective clinical analyses of malaria patients treated with quinolines in these regions would be quite informative concerning the potential use of heme crystallization inhibitors as anti-parasitic agents for both diseases.

## Supporting Information

Figure S1 Dose-dependent inhibition of *S. mansoni* regurgitant-driven Hz formation in vitro by QN, QND and QCR. Representative IC_50_ curves of inhibition of Hz formation in reactions promoted by *S. mansoni* female regurgitants were conducted in the presence of different concentrations of (A) QN, (B) QND and (C) QCR, as described in the methods section. All drugs were tested in concentrations ranging from 5–100 µM.
(7.16 MB TIF)

Figure S2 QN and QND treatment were not toxic to *S. mansoni*-infected mice. Alanine aminotransferase (ALT) (A and C) and aspartate aminotransferase (AST) (B and D) activities were assayed in plasma samples from C, QN (A, B) or QND (C, D) treated mice infected with *S. mansoni* as markers for hepatocellular damage. QN treatment means *S. mansoni*-infected mice treated with 75 mg/kg/day QN from day 11 to 17 after infection, whereas in QND treatment mice were treated from day 42 to 45 after infection with daily intraperitoneal injections of 100 mg/kg QND. Results were expressed as mean±SEM (n = 10, for A and B; n = 3, for C and D).
(4.14 MB TIF)

Figure S3 QN caused a significant reduction in the viability of cultured in vitro transformed schistosomula. Effect of QN treatment on the viability of cultured in vitro-transformed schistosomula assessed by MTT reduction. Control means schistosomula treated with 0.1% ethanol, whereas in the experimental groups, QN was added in concentrations ranging from 10–100 µM. Results were expressed as mean±SEM. * p<0.001, QN 10–100 µM vs. control (C) (one-way ANOVA and a posteriori Tukey's test).
(1.30 MB TIF)

Figure S4 QND treatment later on infection did not affect parasite burden, viability or Hz content. (A) Effect of QND treatment on total number of *S. mansoni* female and male worms in control (n = 5) and QND-treated (n = 5) mice. (B) Effect of QND treatment on the viability of *S. mansoni* female and male worms in control (n = 5) and QND-treated (n = 5) mice. (C) Hz content in female and male *S. mansoni* worms in control (n = 5) and QND-treated (n = 5) mice. Hz was extracted from *S. mansoni* and quantified as described in materials and methods. Control (C): *S. mansoni*-infected mice treated with about 100 µL of 30.0% ethanol. QND: *S. mansoni*-infected mice treated with 100 mg/kg/day QND from day 42 to 45 after infection. Results are expressed as mean±SEM.
(3.35 MB TIF)

Figure S5 QN and QND treatment did not affect protein content in adult worms. (A) Effect of QN treatment on protein content in female and male *S. mansoni* worms. Results are expressed as mean±SEM (n = 27). (B) Effect of QND and QCR treatment on protein content in female and male *S. mansoni* worms (n = 4–9). Control (C) means *S. mansoni*-infected mice treated with about 100 µL of 30.0% ethanol. QN, QND and QCR mean *S. mansoni*-infected mice treated with 75 mg/kg/day of each compound from day 11 to 17 after infection. Results were expressed as mean±SEM. * p<0.001 one-way ANOVA and a posteriori Tukey's test, for males vs. their respective female groups.
(2.50 MB TIF)

Figure S6 QN treatment did not affect total thiol levels nor induce lipid peroxidation in adult worms. (A) Effect of QN treatment on the total thiol content in female and male *S. mansoni* worms (n = 27). (B) Effect of QN treatment on lipid peroxidation in female and male *S. mansoni* worms (n = 12), assessed by the TBARS method. Control (C) means *S. mansoni*-infected mice treated with about 100 µL of 30.0% ethanol, whereas QN means *S. mansoni*-infected mice treated with 75 mg/kg/day QN from day 11 to 17 after infection. Results are expressed as mean±SEM.
(2.41 MB TIF)

Figure S7 QN did not increase reactive species formation in adult worms. Effect of QN treatment on the reactive species formation in female (A) and male (B) *S. mansoni* worms. Intracellular reactive species from *S. mansoni* worms were quantified as described in methods section using the fluorescent probe CMH_2_-DCFDA. Control means *S. mansoni*-infected mice with about 100 µL of 30.0% ethanol, whereas QN means *S. mansoni*-infected mice treated with 75 mg/kg/day QN from day 11 to 17 after infection. Images of worms were acquired in both bright field (left) and epifluorescence (right) microscopy.
(8.43 MB TIF)

Figure S8 QN treatment did not cause ultrastructural changes in the tegument of adult worms. TEM images of tegument cross sections from females (A, B) and males (C, D) of *S. mansoni*. Panels A and C were from control worm, while panels B and D were from QN-treated worm. T- tegument and MF- muscle fibers. Bars denote the scale in micrometers.
(9.60 MB TIF)

Figure S9 QN treatment promotes mitochondrial autophagy and morphological changes in female worms. TEM of cross-sections images from *S. mansoni* adult females obtained from QN-treated mice. (A) Gastrodermis of QN-treated female worm showing a mitochondrion inside of an autophagic vacuole depicted inside the white-dashed box. Panels B and C were from sub-tegumentar region of a female worm obtained from control (B) and QN-treated mice (C). MF-muscular fiber. Arrows indicate a clear swelling of the mitochondria and arrowhead indicates remnants of inner mitochondrial membrane. The asterisk indicates a washed-out mitochondrial matrix and the inset in panel C depicts a magnification of a swollen mitochondrion. Control means *S. mansoni*-infected mice treated with about 100 µL of 30.0% ethanol, whereas QN means *S. mansoni*-infected mice treated with 75 mg/kg/day QN from day 11 to 17 after infection. Bars denote the scale in micrometers.
(9.43 MB TIF)

Figure S10 QN treatment changed gene expression of *S. mansoni* female worms. Genes identified as differentially up regulated in female worms treated with quinine when compared with control females (see methods for details). Heat map representing the 25 genes identified as significantly (FDR 0.1%) over-expressed after treatment. Each line represents one gene and each two adjacent columns represent replicas of one experiment, as indicated at the bottom of the panel. Expression levels of genes are represented by the log2 (treatment/control ratio). Sample 1.x and 2.x represent the dye swap replicates for sample 1 and 2, respectively.
(0.82 MB TIF)
