# Supplementary figures and images for: Interference with Hemozoin Formation Represents an Important Mechanism of Schistosomicidal Action of Antimalarial Quinoline Methanols
Source: PLoS Negl Trop Dis. 2009 Jul 14;3(7):e477. doi: 10.1371/journal.pntd.0000477 (PMC2703804; doi:10.1371/journal.pntd.0000477)

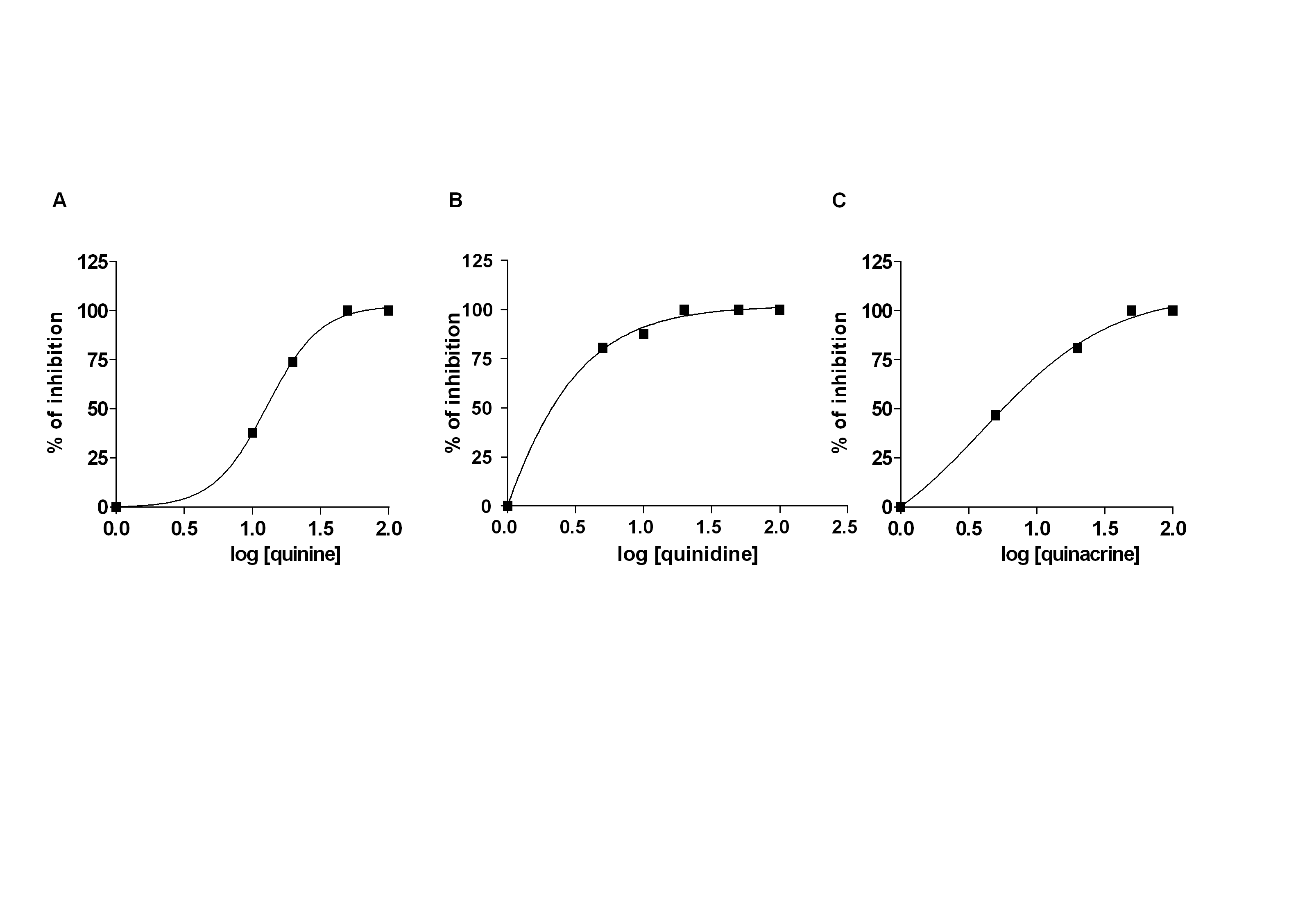

Supplement: Figure S1 — Dose-dependent inhibition of S. mansoni regurgitant-driven Hz formation in vitro by QN, QND and QCR. Representative IC50 curves of inhibition of Hz formation in reactions promoted by S. mansoni female regurgitants were conducted in the presence of different concentrations of (A) QN, (B) QND and (C) QCR, as described in the methods section. All drugs were tested in concentrations ranging from 5–100 µM. (7.16 MB TIF) [file pntd.0000477.s001.tif]

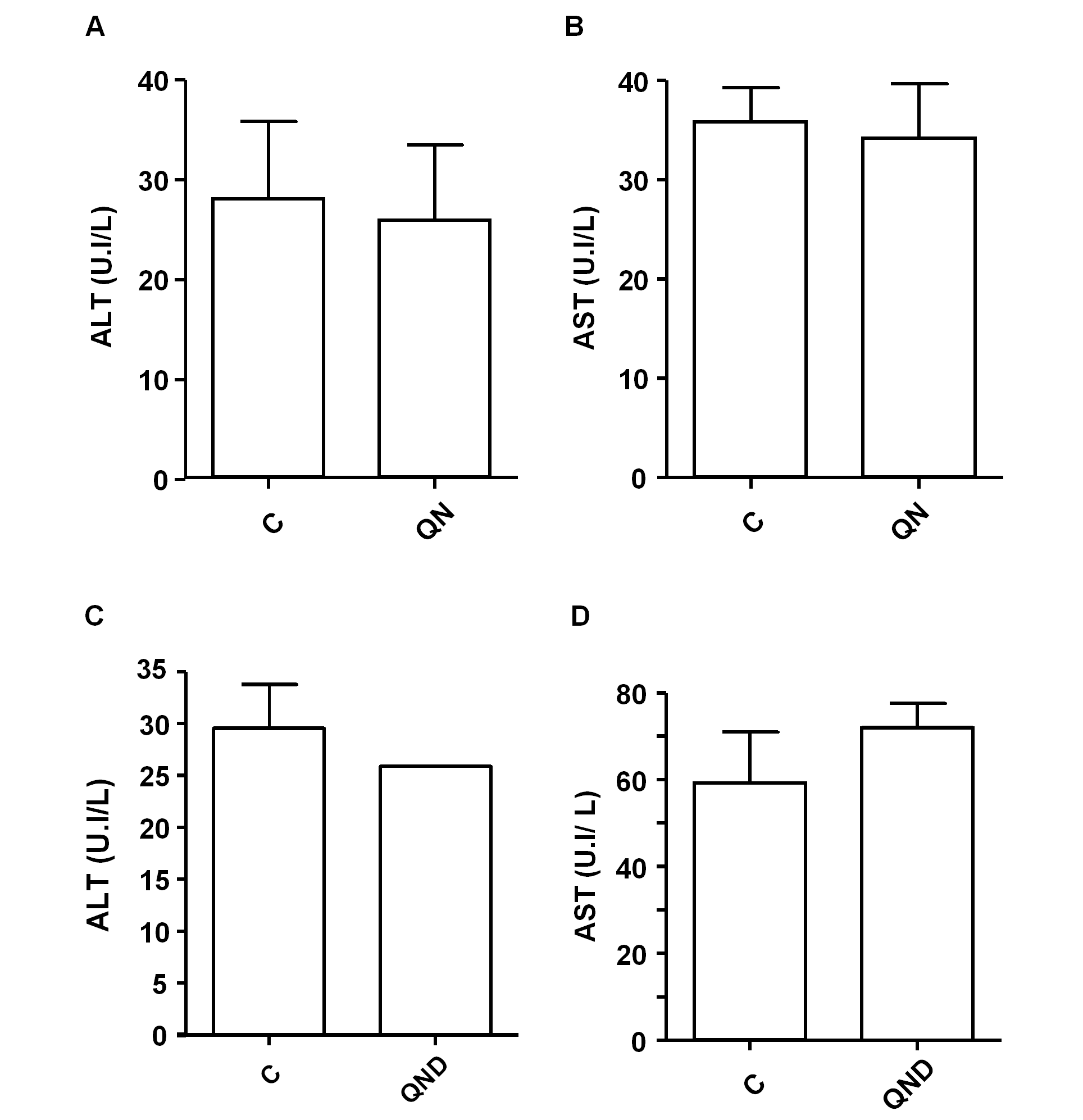

Supplement: Figure S2 — QN and QND treatment were not toxic to S. mansoni-infected mice. Alanine aminotransferase (ALT) (A and C) and aspartate aminotransferase (AST) (B and D) activities were assayed in plasma samples from C, QN (A, B) or QND (C, D) treated mice infected with S. mansoni as markers for hepatocellular damage. QN treatment means S. mansoni-infected mice treated with 75 mg/kg/day QN from day 11 to 17 after infection, whereas in QND treatment mice were treated from day 42 to 45 after infection with daily intraperitoneal injections of 100 mg/kg QND. Results were expressed as mean±SEM (n = 10, for A and B; n = 3, for C and D). (4.14 MB TIF) [file pntd.0000477.s002.tif]

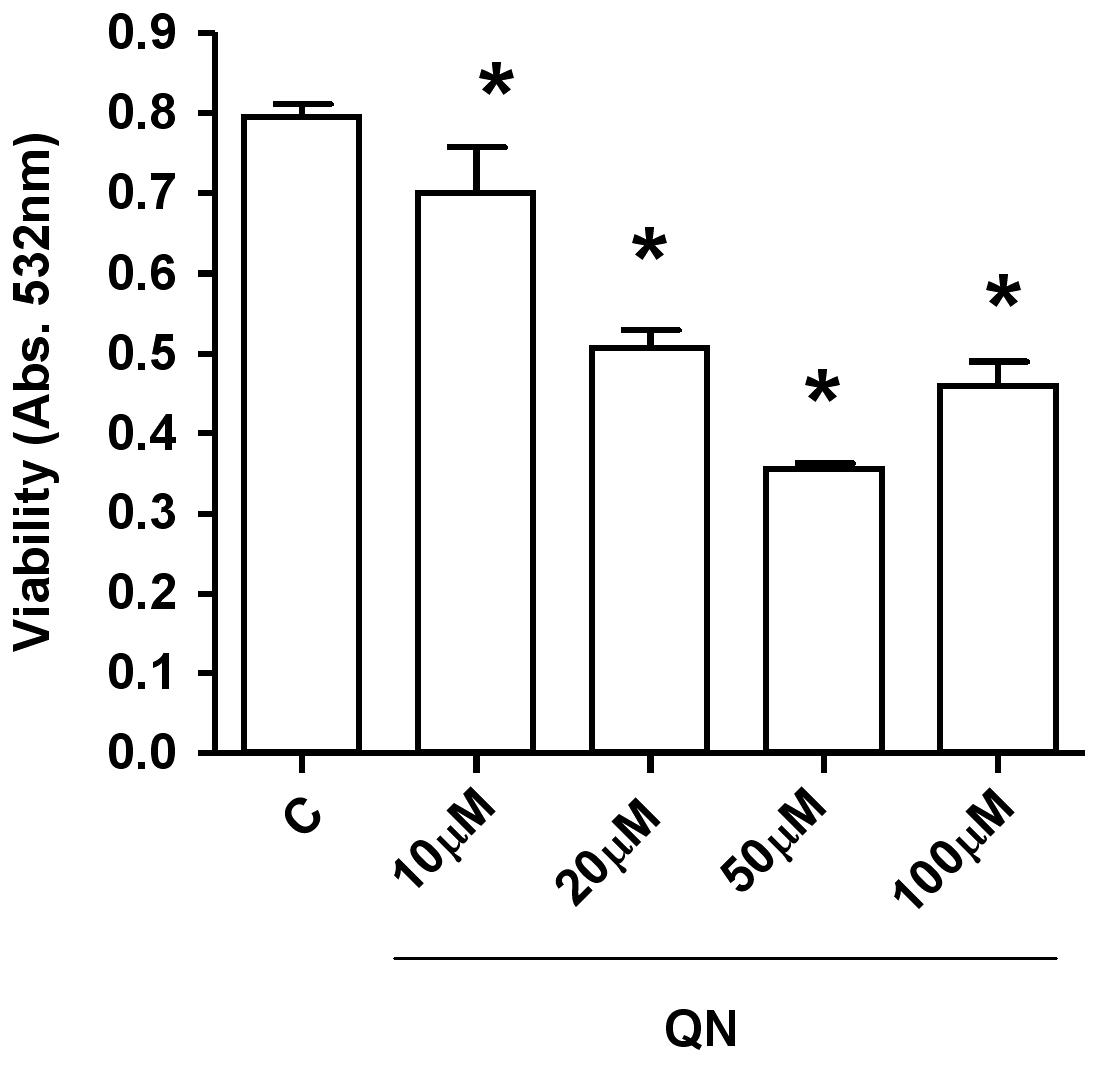

Supplement: Figure S3 — QN caused a significant reduction in the viability of cultured in vitro transformed schistosomula. Effect of QN treatment on the viability of cultured in vitro-transformed schistosomula assessed by MTT reduction. Control means schistosomula treated with 0.1% ethanol, whereas in the experimental groups, QN was added in concentrations ranging from 10–100 µM. Results were expressed as mean±SEM. * p<0.001, QN 10–100 µM vs. control (C) (one-way ANOVA and a posteriori Tukey's test). (1.30 MB TIF) [file pntd.0000477.s003.tif]

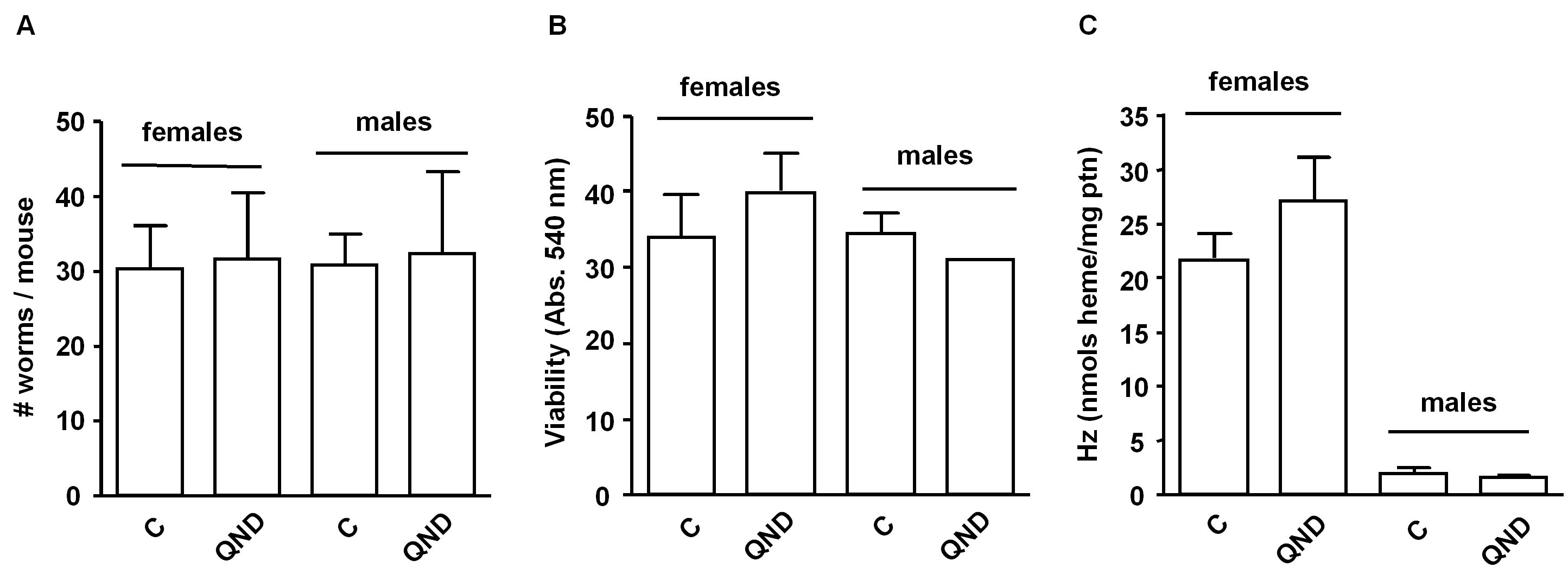

Supplement: Figure S4 — QND treatment later on infection did not affect parasite burden, viability or Hz content. (A) Effect of QND treatment on total number of S. mansoni female and male worms in control (n = 5) and QND-treated (n = 5) mice. (B) Effect of QND treatment on the viability of S. mansoni female and male worms in control (n = 5) and QND-treated (n = 5) mice. (C) Hz content in female and male S. mansoni worms in control (n = 5) and QND-treated (n = 5) mice. Hz was extracted from S. mansoni and quantified as described in materials and methods. Control (C): S. mansoni-infected mice treated with about 100 µL of 30.0% ethanol. QND: S. mansoni-infected mice treated with 100 mg/kg/day QND from day 42 to 45 after infection. Results are expressed as mean±SEM. (3.35 MB TIF) [file pntd.0000477.s004.tif]

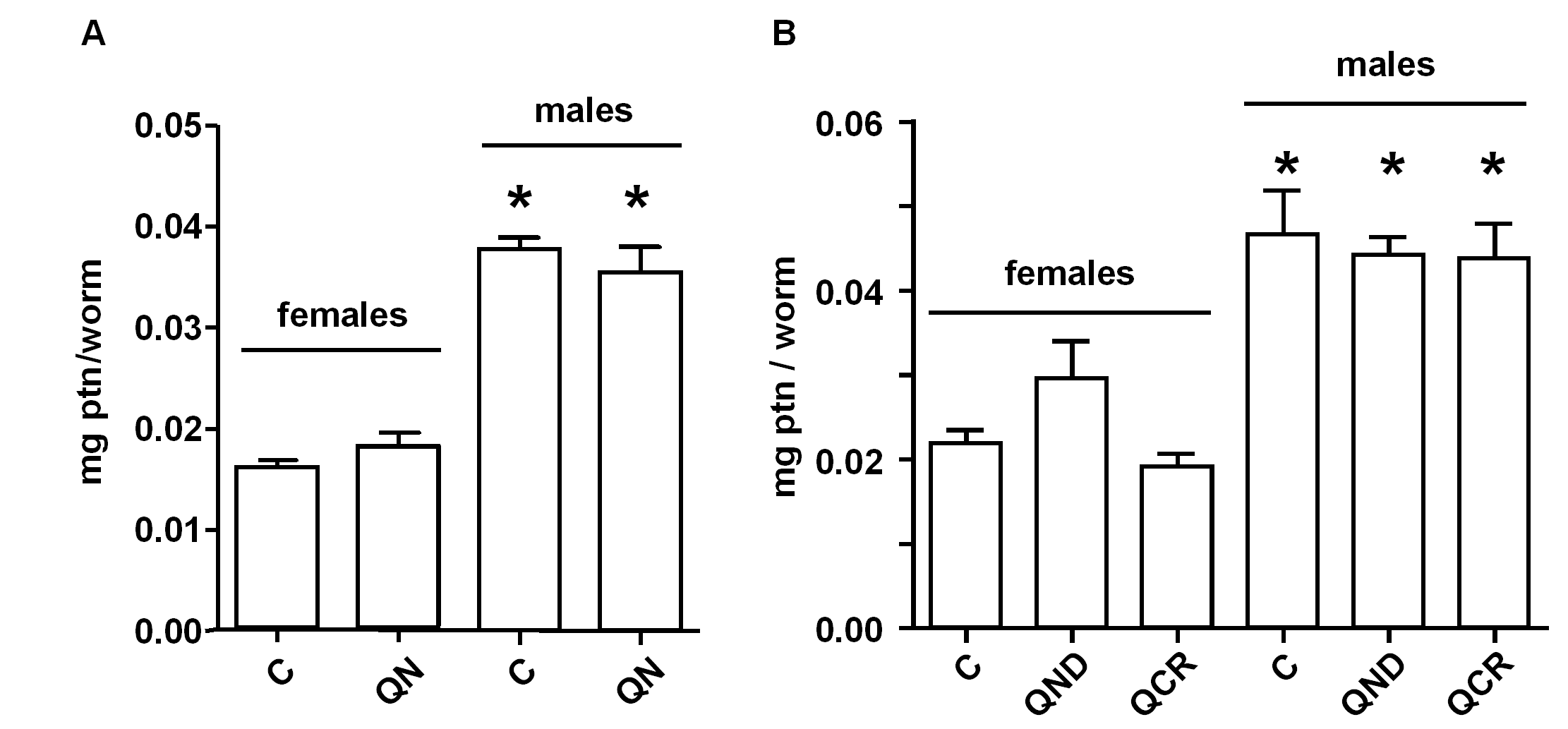

Supplement: Figure S5 — QN and QND treatment did not affect protein content in adult worms. (A) Effect of QN treatment on protein content in female and male S. mansoni worms. Results are expressed as mean±SEM (n = 27). (B) Effect of QND and QCR treatment on protein content in female and male S. mansoni worms (n = 4–9). Control (C) means S. mansoni-infected mice treated with about 100 µL of 30.0% ethanol. QN, QND and QCR mean S. mansoni-infected mice treated with 75 mg/kg/day of each compound from day 11 to 17 after infection. Results were expressed as mean±SEM. * p<0.001 one-way ANOVA and a posteriori Tukey's test, for males vs. their respective female groups. (2.50 MB TIF) [file pntd.0000477.s005.tif]

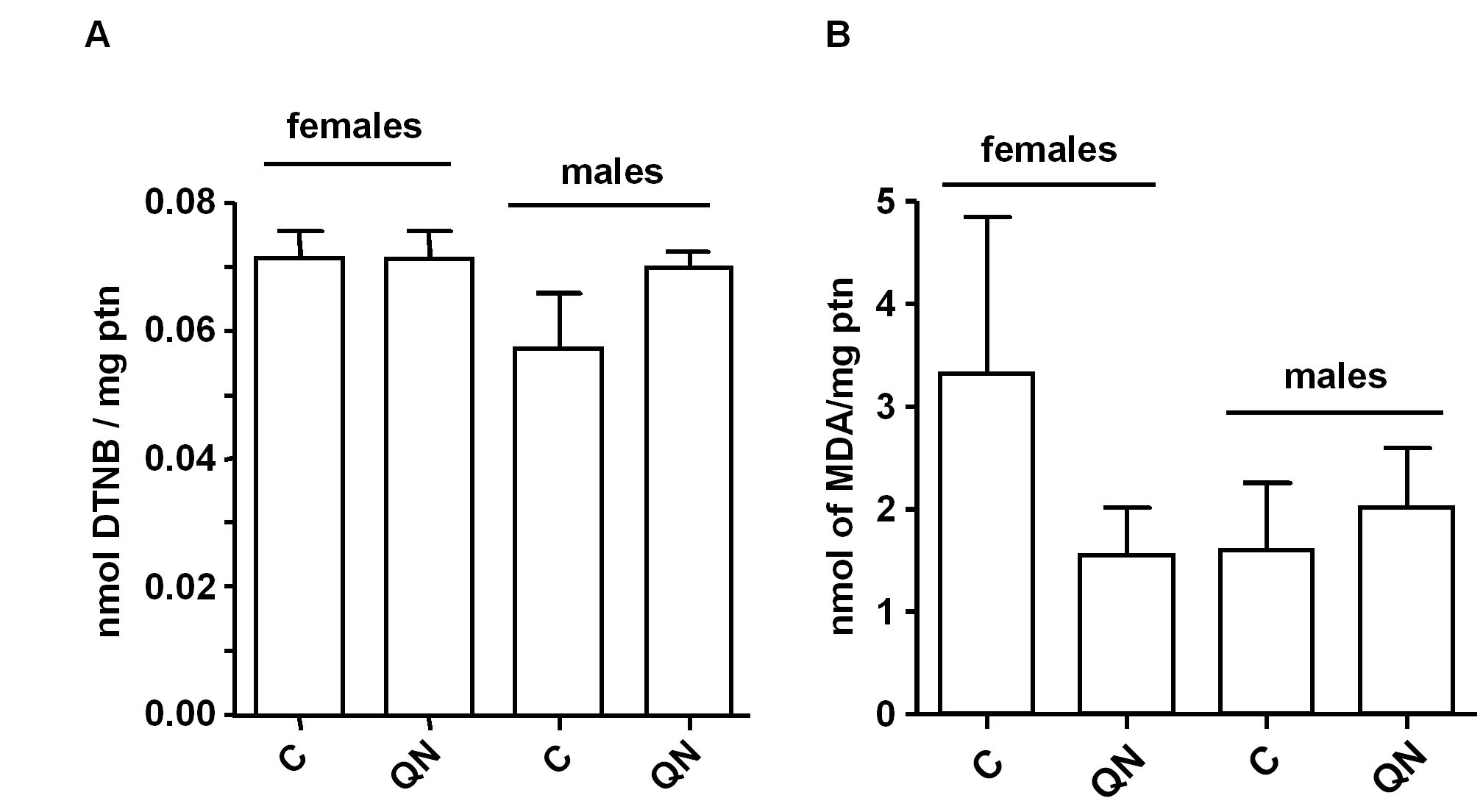

Supplement: Figure S6 — QN treatment did not affect total thiol levels nor induce lipid peroxidation in adult worms. (A) Effect of QN treatment on the total thiol content in female and male S. mansoni worms (n = 27). (B) Effect of QN treatment on lipid peroxidation in female and male S. mansoni worms (n = 12), assessed by the TBARS method. Control (C) means S. mansoni-infected mice treated with about 100 µL of 30.0% ethanol, whereas QN means S. mansoni-infected mice treated with 75 mg/kg/day QN from day 11 to 17 after infection. Results are expressed as mean±SEM. (2.41 MB TIF) [file pntd.0000477.s006.tif]

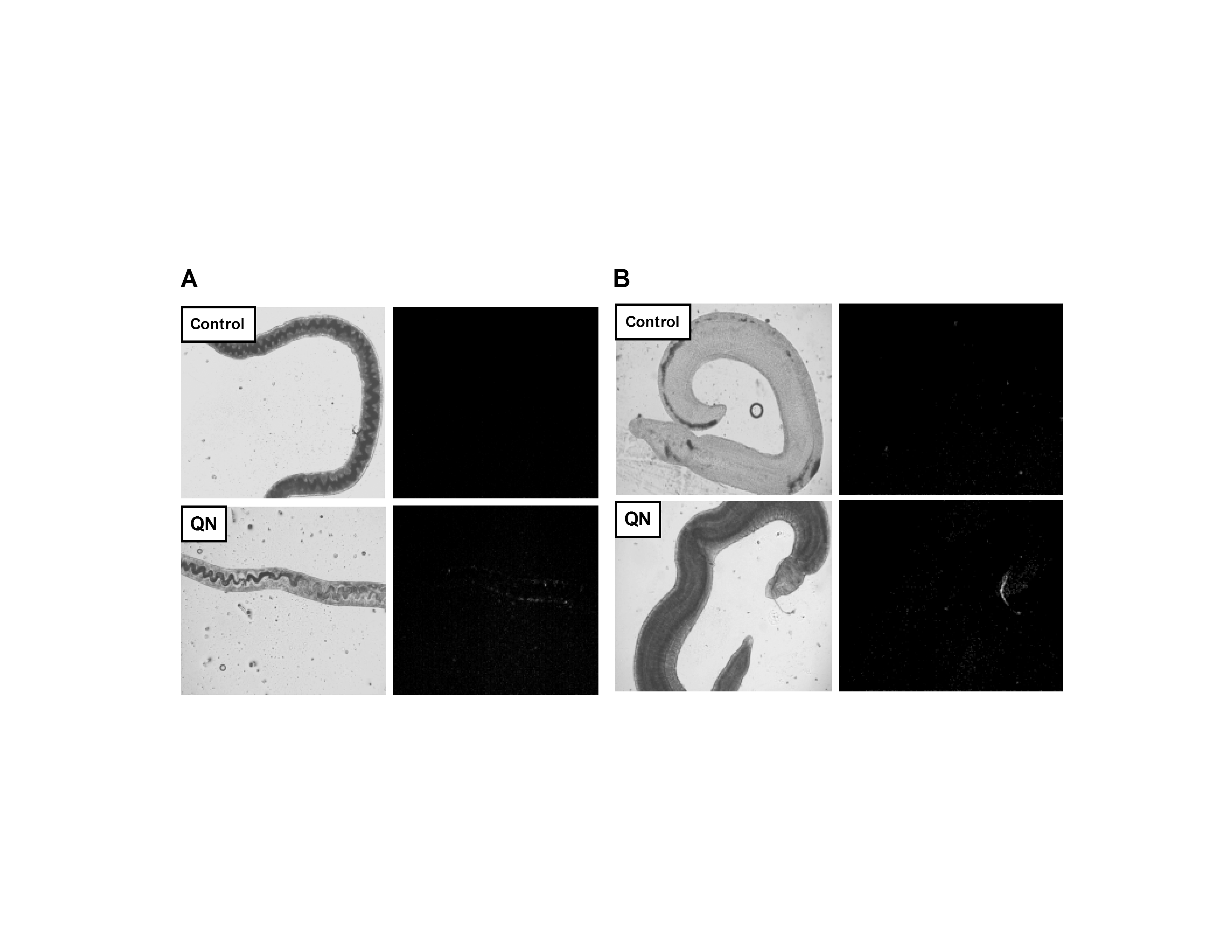

Supplement: Figure S7 — QN did not increase reactive species formation in adult worms. Effect of QN treatment on the reactive species formation in female (A) and male (B) S. mansoni worms. Intracellular reactive species from S. mansoni worms were quantified as described in methods section using the fluorescent probe CMH2-DCFDA. Control means S. mansoni-infected mice with about 100 µL of 30.0% ethanol, whereas QN means S. mansoni-infected mice treated with 75 mg/kg/day QN from day 11 to 17 after infection. Images of worms were acquired in both bright field (left) and epifluorescence (right) microscopy. (8.43 MB TIF) [file pntd.0000477.s007.tif]

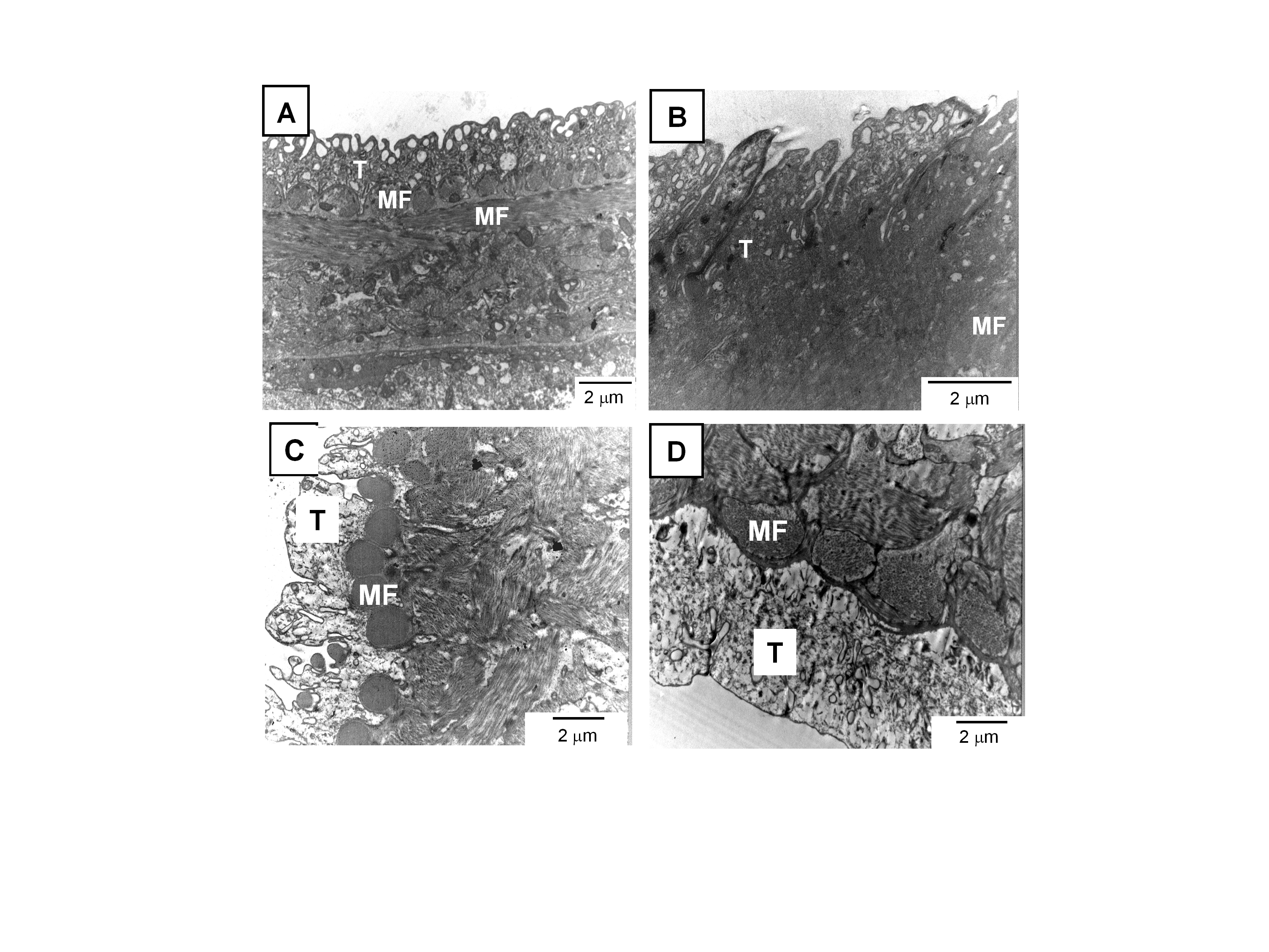

Supplement: Figure S8 — QN treatment did not cause ultrastructural changes in the tegument of adult worms. TEM images of tegument cross sections from females (A, B) and males (C, D) of S. mansoni. Panels A and C were from control worm, while panels B and D were from QN-treated worm. T- tegument and MF- muscle fibers. Bars denote the scale in micrometers. (9.60 MB TIF) [file pntd.0000477.s008.tif]

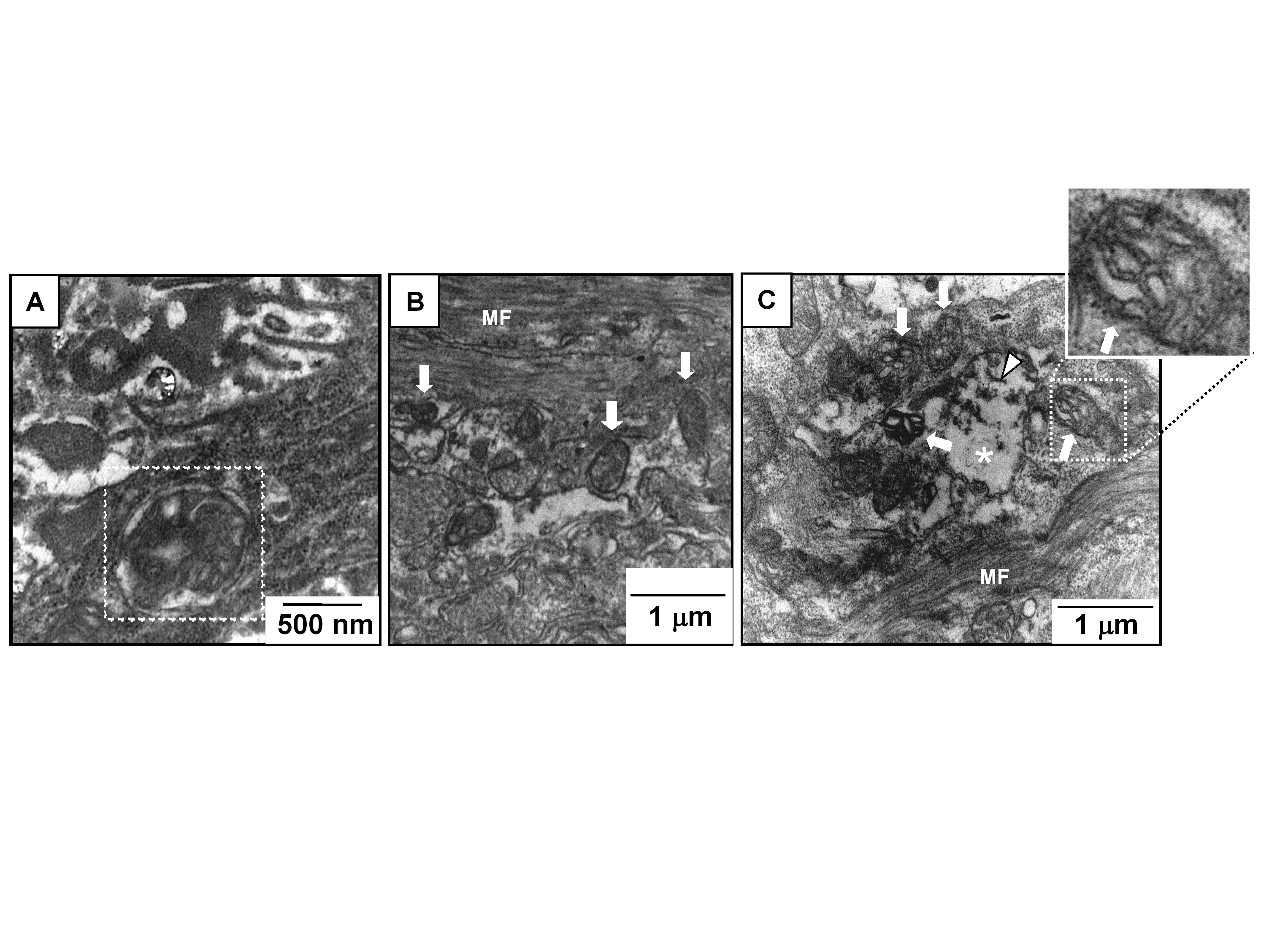

Supplement: Figure S9 — QN treatment promotes mitochondrial autophagy and morphological changes in female worms. TEM of cross-sections images from S. mansoni adult females obtained from QN-treated mice. (A) Gastrodermis of QN-treated female worm showing a mitochondrion inside of an autophagic vacuole depicted inside the white-dashed box. Panels B and C were from sub-tegumentar region of a female worm obtained from control (B) and QN-treated mice (C). MF-muscular fiber. Arrows indicate a clear swelling of the mitochondria and arrowhead indicates remnants of inner mitochondrial membrane. The asterisk indicates a washed-out mitochondrial matrix and the inset in panel C depicts a magnification of a swollen mitochondrion. Control means S. mansoni-infected mice treated with about 100 µL of 30.0% ethanol, whereas QN means S. mansoni-infected mice treated with 75 mg/kg/day QN from day 11 to 17 after infection. Bars denote the scale in micrometers. (9.43 MB TIF) [file pntd.0000477.s009.tif]

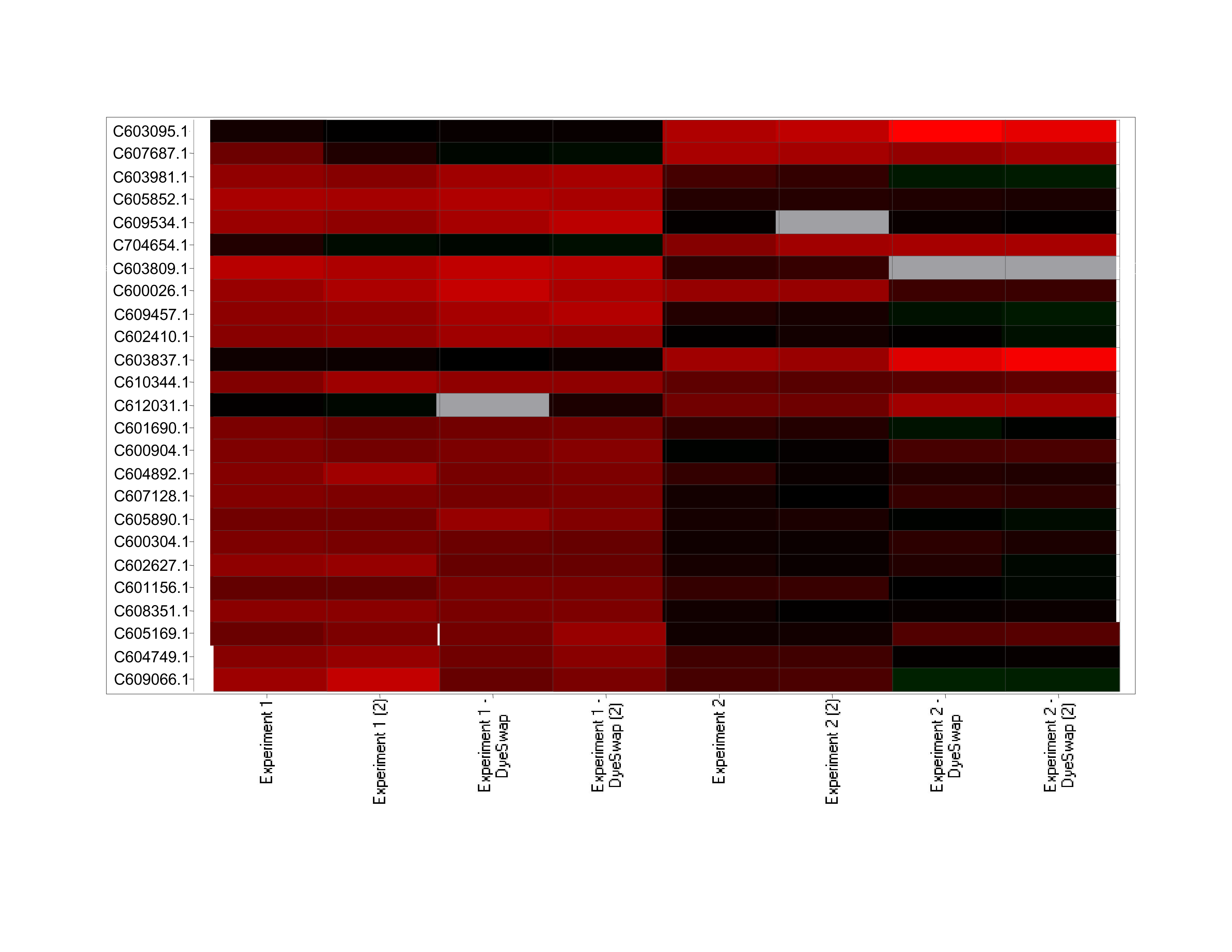

Supplement: Figure S10 — QN treatment changed gene expression of S. mansoni female worms. Genes identified as differentially up regulated in female worms treated with quinine when compared with control females (see methods for details). Heat map representing the 25 genes identified as significantly (FDR 0.1%) over-expressed after treatment. Each line represents one gene and each two adjacent columns represent replicas of one experiment, as indicated at the bottom of the panel. Expression levels of genes are represented by the log2 (treatment/control ratio). Sample 1.x and 2.x represent the dye swap replicates for sample 1 and 2, respectively. (0.82 MB TIF) [file pntd.0000477.s010.tif]
